# Red blood cell-activated antifreeze metal-organic framework armor for cryopreservation

**DOI:** 10.1016/j.mtbio.2025.102529

**Published:** 2025-11-07

**Authors:** Xin Li, Zhaoyang Gong, Muzhe Xu, Song Wang, Bingbing Sun

**Affiliations:** a State Key Laboratory of Fine Chemicals, Dalian University of Technology, 2 Linggong Road, 116024, Dalian, China; b School of Chemical Engineering, Dalian University of Technology, 2 Linggong Road, 116024, Dalian, China; c Frontiers Science Center for Smart Materials Oriented Chemical Engineering, School of Chemical Engineering, Dalian University of Technology, 2 Linggong Road, 116024, Dalian, China; d State Key Laboratory of Mesoscience and Engineering, Institute of Process Engineering, Chinese Academy of Sciences, Beijing, 100190, China

**Keywords:** Ice recrystallization inhibition, Armored structure, Nano-bio interface, Metal-organic frameworks, Cryopreservation

## Abstract

Red blood cell (RBC) cryopreservation is essential for maintaining ex vivo biological function and enabling long-term storage. However, the fragile nature of RBCs makes them highly susceptible to hemolysis during freezing and thaw cycles, limiting their widespread application. Here, a metal-organic framework (MOF)-based armor has been developed in a physiological environment, which enables one-step in situ encapsulation and unloading of RBCs. This MOF armor, functioning as a nano-bio interface, effectively shields RBCs from cryo-induced damage while preserving their native oxygen-carrying function. The MOF-armored RBCs not only inhibit ice recrystallization by restricting water molecule movement within the porous MOF structure but also serve as a “catalyst” to induce the smoothing of the ice front during growth, reducing sodium chloride eutectic formation and accelerating ice melting. Cryopreservation assays demonstrate exceptional RBC recovery (51 %) at a low MOF concentration (1 mg mL^−1^), confirming the effectiveness of this protective strategy in enhancing cryoprotectant efficacy. These findings highlight the potential of MOF-derived architectures for the cryopreservation of various cell types and tissue samples, paving the way for innovative preservation technologies.

## Graphical abstract

A MOF-based armor enables the in situ encapsulation and unloading of RBCs, effectively protecting them from freeze-thaw damage. This protection is achieved by reducing ice grain size through the molecular-level restriction of free water molecule mobility, smoothing the ice growth front, and shortening cellular exposure to the ice phase through accelerated ice melting during thawing.

**Figure:**
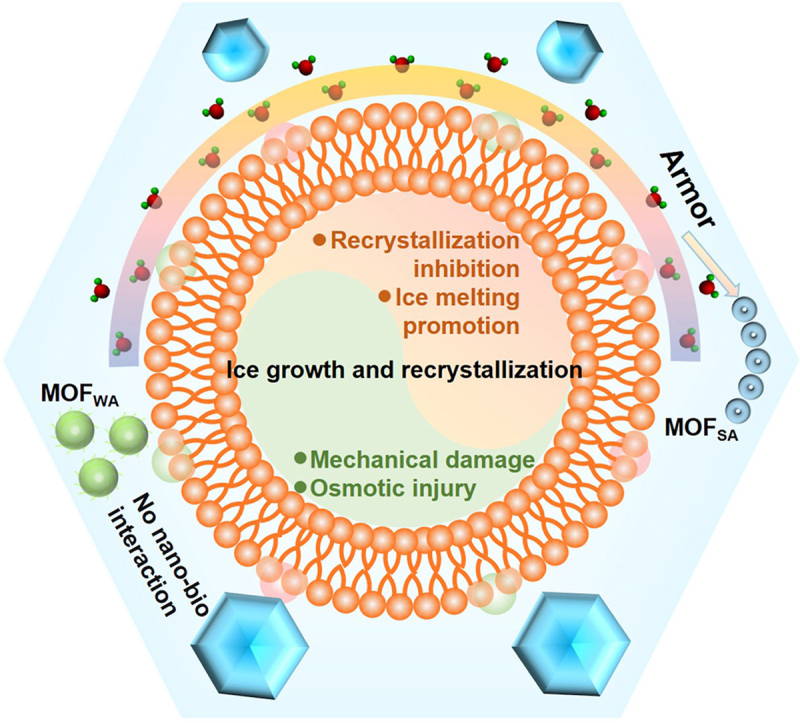

## 1 Introduction

The preservation of red blood cells (RBCs) is essential due to the high demand for RBC products in transfusions and biomedical engineering [1,2]. Cryopreservation, which suspends cellular metabolism at low temperatures (−80 °C or −196 °C), plays a crucial role in maintaining RBC functionality ex vivo and enabling long-term storage, thereby reducing unnecessary waste [3,4]. However, the freezing and thawing processes introduce significant physiological stress, primarily due to damage caused by ice crystals [5,6]. Ice nucleation, growth, and recrystallization cause irreversible cryoinjury, compromising cell integrity and reducing preservation efficiency [7]. Cryoprotectants (CPAs) have driven significant advancements in cryobiology [8]. High concentrations of cell-permeable CPAs, such as dimethyl sulfoxide and glycerol, help minimize ice formation. However, their solvent toxicity and the challenges associated with their removal pose major barriers to clinical applications [9,10]. Inspired by the antifreeze proteins (AFPs) naturally produced in extreme cold environments [11,12], various synthetic compounds and nanomaterials mimicking AFP functions have been explored for cryopreservation. For example, poly (vinyl alcohol) [[13], [14], [15]] and zwitterionic betaine [16,17] enable cell-friendly regulation of ice crystals, and nanoparticles such as graphene oxide and quasi-carbon nitride quantum dots exhibit ice recrystallization inhibition (IRI) properties, making them promising candidates for cell cryopreservation [18,19]. Despite these advancements, developing novel nanomaterials with strong IRI activity and efficiently enhanced RBC recovery remains a highly desirable goal.

Metal-organic frameworks (MOFs) are well-defined porous materials that are typically self-assembled from metal nodes and organic linkers [[20], [21], [22], [23], [24], [25], [26]]. Their highly tunable structures and versatile properties have gained significant attention, enabling applications across diverse fields, including gas storage and separation [27], chemical catalysis [28,29], energy [30], drug delivery [31,32], and biomedical applications [33]. In cryopreservation, Brinker et al. demonstrated the use of zirconium (Zr)-based MOF nanoparticles (NPs) with well-defined surface chemistries for RBC cryopreservation and highlighted hafnium (Hf)-based 2D metal-organic layers for inhibiting ice growth [34,35]. Inspired by the modular and precisely controlled chemistry of MOFs, the concept of “armored red blood cells” (armored RBCs) as a bio/artificial hybrid nanosystem was introduced [[36], [37], [38], [39]]. These RBCs, encapsulated within MOF-based shells, retain their normal oxygen-carrying function while gaining enhanced resistance to external stress. However, their potential as candidates for advanced cryopreservation strategies, such as developing armored RBCs and controlled RBC release, has yet to be realized, and their underlying mechanisms remain unclear.

Therefore, we propose a novel strategy for the cryopreservation of RBCs using zinc (Zn)-based MOFs, which can efficiently encapsulate RBCs into MOFs in a physiological environment. In the sheep RBC cryopreservation test, MOF@RBC demonstrated excellent cell recovery efficiency (∼51 %) at a concentration of 1 mg mL^−1^, matching the performance of the commercial cryoprotectant HES (200 mg mL^−1^). Furthermore, MOF_SA,_ capable of autonomously forming a protective shell, was synthesized. While MOF_SA_ itself does not exhibit IRI activity, the introduction of RBCs activates this property, highlighting the significance of the MOF-armored RBC structure for ice control. These MOF-armored RBCs not only suppress ice crystal growth by restricting the movement of free water molecules but also function as a “catalyst” to smooth the ice front, mitigate sodium chloride eutectic formation, and promote the melting of ice crystals, thereby enhancing cryopreservation efficiency. This armor strategy highlights the potential of MOF-based shell structures for effective cryopreservation, paving the way for broader applications of MOF-inspired architectures in biological applications.

## 2 Experimental section

### 2.1 Materials

Imidazole-2-formaldehyde (ICA) was purchased from Macklin (Shanghai, China). Zinc nitrate hexahydrate was purchased from Xilong (Guangdong, China). Polyvinyl pyrrolidone (PVP) (M.W. 40,000) and hydroxyethyl starch (HES) were purchased from Aladdin (Shanghai, China). Sodium dithionite (Na_2_S_2_O_4_) was purchased from Meryer (Shanghai, China). Luminol and sodium perborate tetrahydrate were purchased from Mreda (Beijing, China). All of the chemicals were used without further purification. Defibrinated sheep blood was purchased from Bikeman (Hunan, China). The preservation and procedures of sheep RBC were approved by the Animal Research Committee at DUT (approval number: DUTSCE250916-02).

### 2.2 Synthesis of MOFs

For the synthesis of MOF_WA_, following the previously reported methods [40], zinc nitrate hexahydrate (3.7 mg) was added to pure water (150 μL) and then mixed with 1000 μL of pure water containing polyvinylpyrrolidone (PVP) (0.5 mg) and imidazole-2-carboxaldehyde (4.8 mg). The resulting mixture was stirred for 10 min at 20 °C. Subsequently, the products were collected by centrifugation (6000 rpm) for 10 min and washed with excess pure water. Finally, they were redispersed in 1150 μL of saline for future use.

For the synthesis of MOF_SA_, the pure water solvent in the synthesis process was replaced with saline, and no other changes were made. A total of 25 μL of RBCs was added to the MOF_SA_ dispersion, and the resulting products were named MOF_SA_ + RBC (for 10 min mixing time) and MOF_SA_ + RBC-S (for 10 s mixing time), based on the duration of the mixing process.

For the synthesis of MOF@RBC, 25 μL of RBCs (5 × 10^8^) were mixed with 1000 μL of saline, to which 0.5 mg of polyvinylpyrrolidone (PVP) and 4.8 mg of imidazole-2-carboxaldehyde were added. Then, another 150 μL of saline was added, in which 3.7 mg of zinc nitrate hexahydrate was dissolved. The resulting mixture was stirred for 10 min at room temperature.

### 2.3 Physicochemical characterizations

Scanning electron microscopy (SEM) analyses and energy-dispersive X-ray spectroscopy (EDS) elemental mappings were performed using a SU88220 field-emission scanning electron microscope (Hitachi, Japan). Transmission electron microscopy (TEM) imaging was conducted using a JEM-1400 Flash transmission electron microscope (JEOL, Japan) to analyze the morphology of NPs. Powder X-ray diffraction (PXRD) patterns were collected using an X-ray diffractometer (Bruker, Germany) with monochromatized Cu Kα radiation (λ = 0.15418 nm). The zeta potentials of NPs in water were measured using a 90-plus PALS dynamic light scattering instrument (Brookhaven, USA). Thermogravimetric analysis (TGA) was conducted by heating NPs from room temperature to 800 °C at a rate of 10 °C·min^−1^ using a TGA 4000 diamond thermogravimetric/differential thermal analyzer (PerkinElmer, USA). The structural properties of NPs were analyzed using an iS50 Fourier transform infrared spectroscopy spectrometer (FTIR, Thermo Fisher, USA). The UV–visible analysis was conducted using a SpectraMax® i3x multi-mode microplate reader (Molecular Devices, USA). Osmotic pressures of solutions were measured using a Vapro5600 osmometer (Wescor, USA). An inductively coupled plasma emission spectrometer (ICP-OES, AVIO 500, USA) was used to measure the concentration of zinc ions.

The NMR spin-spin relaxation time (T_2_) measurements were conducted using a PQ001 low-field NMR spectrometer (Suzhou Numag Analytical Instrument Corporation, China) operating at a proton resonance frequency of 333.33 kHz. T_2_ was calculated using the Carr-Purcell-Meiboom-Gill (CPMG) pulse sequence. The echo time (TE) was 0.8 ms, the number of echoes (NECH) was 18,000, and the waiting time between two repeated samplings (TW) was 15,000 ms. The relaxation time measurements were carried out at 35 °C, and the weighted imaging measurements were performed at 32 °C.

### 2.4 Degradation of nanoparticles

All the NPs were dispersed in a citric acid phosphate buffer (pH 6.25). A stepwise addition of the buffer was employed to minimize cell damage caused by material peeling from the cell membrane. Specifically, 100 μL of citric acid phosphate buffer was added every 5 min until the volume of the buffer added was twice that of the raw material to ensure complete dissolution.

### 2.5 Quartz crystal microbalance with dissipation measurement

A quartz-crystal microbalance with a dissipation instrument (QCM-D, Biolin Scientific AB, FI) was utilized to record changes in resonant frequency (Δf) over time [41]. All measurements were conducted using optically polished gold-deposited quartz crystals. The sensors were prepared as follows: QCM-D sensors were immersed in a mixture of 10 mL of pure water, 2 mL of ammonium hydroxide, and 2 mL of hydrogen peroxide at 75 °C for 5 min. Afterward, the sensors were rinsed with pure water and sonicated for 2 min. Subsequently, cleaned sensors were dried with a gentle stream of nitrogen. The cleaned sensors were then dipped in 50 mM 3-mercaptopropionic acid (3-MPA) overnight. The 3-MPA-modified sensors were thoroughly rinsed in ethanol and pure water and then incubated with poly-L-lysine for 1 h. Afterward, the sensors were rinsed with water, dried, and prepared for cell culture. The sensor was mounted in a flow module (Q-sense), and the saline was injected at room temperature. Following the establishment of stable baselines, the flow was resumed by switching to 4 × 10^5^ cells·mL^−1^ RBC solution. Until the frequency alteration ceases, the flow was altered to a 100 μg mL^−1^ NPs dispersion. Changes in frequency (Δf) are recorded simultaneously until a steady value is achieved.

### 2.6 Adsorption capacity of MOF for various components

Glucose (Glu), phosphatidylcholine (PC), total cholesterol (TC), and hemoglobin (Hb) were chosen to serve as representative models of cell membrane components. These substances were then utilized to evaluate the adsorption between them and MOF NPs. Briefly, a series of samples with concentrations ranging from 5 to 0.1 mg mL^−1^ were prepared and incubated with a fixed concentration of the substance for 10 min. After centrifugation, the remaining substance concentration in the supernatant was measured. The Glu content was quantified using a Glu content detection kit (Solarbio, Beijing, China), and the standard concentration of Glu used for incubation with MOFs was 0.72 mg mL^−1^, diluted 2 times before testing. The PC content was determined using a Sheep PC ELISA kit (Mreda, Beijing, China), and the standard concentration of PC used for incubation with MOFs was 1.8 μg mL^−1^, diluted 5 times before testing. The TC content was quantified with a TC content detection kit (Sangon Biotech, Shanghai, China), and the standard concentration of TC used for incubation with MOFs was 1 mg mL^−1^. The Hb levels were assessed using the Pierce™ BCA protein assay kit (Thermo Scientific, USA), and the standard concentration of Hb used for incubation with MOFs was 1 mg mL^−1^. The adsorption intensity (%) was defined as the percentage decrease from the initial concentration, and the linear ranges of the assays were 0.01–0.45 mg mL^−1^, 0.012–0.5 μg mL^−1^, 0.01–4.83 mg mL^−1^, and 0–2 mg mL^−1^ for Glu, PC, TC, and Hb, respectively.

### 2.7 Hemolysis of RBCs

The purchased sheep RBCs were centrifuged at 300 g for 5 min at 4 °C. Then, the supernatant was replaced with an equal volume of saline (SA) solution. This process was repeated three times to ensure that residual plasma or saccharides were removed thoroughly. To determine hemolysis rates of key precursors, specifically zinc ions (Zn^2+^) and imidazole-2-formaldehyde (ICA), a series of solutions containing zinc nitrate hexahydrate and ICA was prepared, with concentrations spanning from 0.25 to 2 times those specified in the MOF_SA_ armor synthesis protocol. The baseline concentration was set at 1.0, corresponding to a Zn^2+^ concentration of 10.55 mM and an ICA concentration of 42.5 mM. Then 25 μL of RBC suspension was added to each sample. The samples were then incubated at room temperature for 2 h. After incubation, all samples underwent centrifugation at 300*g* for 3 min to separate and remove the intact cells. The absorbance of the resulting supernatant was subsequently measured at 541 nm using a microplate reader. A background control group was established, in which no RBCs were added. The hemolysis rate was calculated using the following equation:

(1) $$Hemolysis\left(\%\right)=\frac{100*\left({\text{OD}}_{541\text{nm}}\left(Sample\right)-{\text{OD}}_{541\text{nm}}\left(\text{background}\right)\right)}{{\text{OD}}_{541\text{nm}}\left(100\%\right)-{\text{OD}}_{541\text{nm}}\left(0\%\right)}$$

Here, OD_541nm_ (0 %) and OD_541nm_ (100 %) represent the absorbances of fresh RBCs and 100 % lysed RBCs, respectively.

### 2.8 Biocompatibility and cryopreservation assay of RBCs

For the biocompatibility assay, all samples were gently vortexed and stored in a refrigerator at 4 °C for 24 h before measuring RBC cell recovery. For the cryopreservation of RBCs, two different freeze-thawing protocols were used. The first method involved cooling cryotubes containing the prepared samples to −80 °C for 24 h, followed by thawing in a water bath at 37 °C. The second procedure involved immersing each sample in liquid nitrogen for 2 h. Subsequently, the samples were allowed to thaw at 4 °C for 150 min before measuring cell recoveries. For comparison, the commercial cryoprotectant HES was also dissolved in saline (200 mg mL^−1^), and the freeze-thawing process described above was repeated.

The MOF armor in the MOF@RBC and MOF_SA_ + RBC was removed by the citric acid stepwise degradation method, and the remaining sample was then adjusted to an equal volume by adding saline. All samples were centrifuged at 300 g for 3 min to remove the intact cells. Subsequently, the absorbance of the supernatant at 541 nm was measured using a microplate reader to assess the extent of hemolysis and measure cell recovery. Samples with 0 % hemolysis were prepared by adding saline to 25 μL of the prepared RBC dispersion and leaving them untreated in the same environment as the biocompatibility/cryopreservation experiments. 100 % hemolysis samples were prepared by adding Triton-X100 to 0 % hemolysis samples and subjecting them to sonication. Note that for each batch experiment, both 100 % and 0 % hemolysis samples were freshly prepared. Cell recovery (%) was calculated by the equation below

(2) $$\text{Cell}\;\text{recovery}\;\left(\%\right)=\frac{100*\left({\text{OD}}_{541\text{nm}}\left(100\%\right)-{\text{OD}}_{541\text{nm}}\left(\text{Sample}\right)\right)}{{\text{OD}}_{541\text{nm}}\left(100\%\right)-{\text{OD}}_{541\text{nm}}\left(0\%\right)}$$

### 2.9 Membrane osmotic shock-resistance capability assays

The membrane osmotic shock-resistance of RBCs was determined by measuring their hemolysis rates in solutions with NaCl concentrations from 0.0 % to 1.2 %. The procedures and viability calculation methods are the same as described in Part 2.7 above.

### 2.10 Assessment of RBC deformability

A homemade negative-pressure microporous filtration device was employed. A stable negative-pressure environment was established by aspirating 10 mL of air from the system using a syringe. The top of the liquid storage chamber remained open to atmospheric pressure. A digital stopwatch was used to measure the time required for the sample volume to traverse a 3 μm pore-sized filter membrane. Reduced cellular deformability was expected to prolong filtration time due to increased resistance to membrane passage.

### 2.11 Phosphatidylserine externalization assays

To evaluate phosphatidylserine (PS) externalization, fluorescence-labeled Annexin V (FITC-Annexin V) staining was used due to its high affinity for PS exposed on the outer membrane. FITC-Annexin V and binding buffer were prepared according to the manufacturer's instructions of the Annexin V FITC Apoptosis Detection Kit (Beyotime, China). After washing the RBCs once with saline, they were resuspended in binding buffer to a final concentration of 2.0 × 10^6^ cells/mL, and then 195 μL of this RBC suspension (containing 3.9 × 10^5^ cells) was incubated with 5 μL of Annexin V-FITC for 10 min at room temperature in the dark. The level of PS externalization was determined by flow cytometry (BD FACSymphony AI, USA), with each sample containing 1.0 × 10^4^ cells, and the data were analyzed using FlowJo V10 software.

### 2.12 Relative adenosine triphosphate (ATP) level assays

An ATP Detection Kit (Beyotime, China) was used to determine the ATP levels. Native RBCs and post-thawed RBCs, each with 1.0 × 10^8^ cells/mL, were suspended in lysis buffer. All samples were centrifuged at 12,000 g for 5 min. Then, 100 μL of working buffer was added to each well of a 96-well plate, followed by the supernatant of the native and post-thawed RBCs, which were fully lysed. The mixture was gently mixed, and the luminescence intensity was measured using a microplate reader. The ATP level of the fresh RBC group was designated as 100 %.

### 2.13 Chemiluminescence measurement

Luminol-based chemiluminescence was utilized to evaluate the oxygen-carrying capacity of RBCs [42]. Briefly, 250 mg of sodium carbonate, 35 mg of sodium perborate, and 5 mg of luminol were dissolved in 5 mL of water. The luminol solution was allowed to stand undisturbed for 5 min in a dark room. Subsequently, 150 μL of samples in saline were added to black 96-well plates at 100 million cells·mL^−1^. Then, 30 μL of luminol solution was introduced to each well, and the contents were mixed for 30 s in the dark. The luminescence intensity trends of the samples over time were measured using a microplate reader.

### 2.14 Determining the capability for reversible oxygen binding

The ability to reversibly bind oxygen was evaluated by analyzing changes in the UV–Vis absorption spectra (350–650 nm) of oxygenated and deoxygenated solutions [43]. To achieve complete deoxygenation, nitrogen gas was bubbled through the sample solution to eliminate most of the oxygen. After 10 min, Na_2_S_2_O_4_ was added to a final concentration of 0.2 mg mL^−1^, and the UV–Vis absorption spectrum was scanned using a microplate reader. For reoxygenation, the sample solutions were exposed to atmospheric oxygen for over 1 h, and the UV–Vis absorption spectrum was recorded. This procedure represents the standard method for assessing the ability to bind oxygen reversibly.

### 2.15 Determination of ice recrystallization inhibition (IRI) activity

The IRI activity was evaluated using a typical splat cooling method [18]. An ECLIPSE LV100N POL polarized optical microscope (Nikon, Japan) and a BCS196 cooling stage (Linkam, UK) were used for this investigation. Specifically, from a height of 1.5 m above the slide, 15 μL of dispersion at room temperature (25 °C) was dropped onto a slide pre-cooled to −60 °C, forming a thin solid ice film. The temperature of the ice film was then increased to −9 °C at a rate of 10 °C·min^−1^ and annealed at this temperature for 30 min to assess the IRI activity. Subsequently, three independent ice wafers were imaged to determine the maximum grain size (MGS) of the ice crystals. The MGS was defined as the straight-line distance between the two farthest vertices of an ice crystal. Significant visible ice crystal particles in the fields of view were measured. After data sorting, the five largest ice grain sizes from each ice wafer were selected to evaluate the IRI activity using AxioVision software.

### 2.16 Determining the critical rate associated with the curvature transition of the ice growth front and the formation of single crystals

A homemade miniature pool was used to determine the critical rate associated with the morphological transition of the advancing ice front. In summary, the right side of the unit was positioned in the center of the Linkman cooling stage to locally reduce the temperature, while the other side remained at ambient temperature at 15 °C. This setup spatially induced a gradual temperature change from microtherm to ambient across a length of 2.5 cm (from right to left). The saline solution, with and without MOF particles confined within the microfluidic channel mounted onto this apparatus, undergoes a gradual growth of crystalline ice from microtherm to ambient areas, with growth slowing progressively away from the cold source. Ice front morphology transitions, from sharp to smooth, were measured at the initial and ending times to calculate critical transition velocities. The curvature was obtained by ImageJ. To determine the growth of single ice crystals, 1 μL of NPs dispersion was added to the center of a glass coverslip, and a second coverslip was placed on top to create a thin film of liquid between the two coverslips. The dispersion was quickly frozen to −20 °C and then gradually warmed to the melting temperature. Once a small single ice crystal appeared, the temperature was further decreased at a rate below 0.2 °C·min^−1^ to facilitate ice crystal growth.

### 2.17 Ice melting analysis

The heat flow changes during the ice melting process of NPs dispersion were recorded using an Optical Linkman cooling stage (DSC450, UK). The samples were cooled from 25 °C at a rate of 20 °C·min^−1^ to −60 °C, with the sample mass controlled at 10 mg. They were held for 3 min and then rewarmed to 20 °C at a slower rate of 2 °C·min^−1^.

### 2.18 Statistical analysis

Results were shown as mean ± standard deviation. One-way ANOVA followed by Tukey's post-hoc test was used for multiple comparisons, and a two-tailed Student's t-test was used for comparison between two groups.

## 3 Results and discussion

### 3.1 Synthesis of MOF-armored RBCs in physiological conditions

**Fig. 1:**
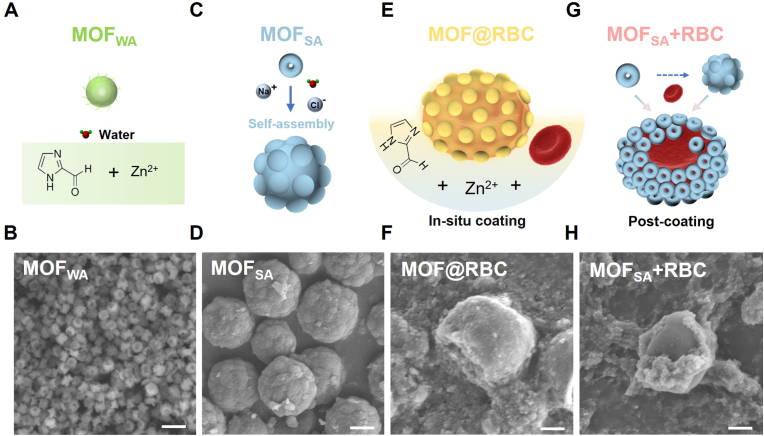
Schematic demonstration and characterization of MOF-armored RBCs. (A) Schematic and (B) SEM image of MOF_WA_, synthesized with 12.4 μmol of Zn^2+^ (Zn^2+^: ICA molar ratio of 1:4) in water. (C) Schematic and (D) SEM image of MOF_SA_, generated in saline. (E) Schematic and (F) SEM images of MOF@RBC, formed by in situ loading of 5 × 10^8^ RBCs into MOF armor, forming spore-like structures efficiently. (G) Schematic and (H) SEM images of MOF_SA_ + RBC, produced by physically mixing pre-synthesized MOF_SA_ and RBCs for 10 min. The scale bar is 1 μm.

A facile strategy for synthesizing MOFs under aqueous conditions without pressurization or heating was employed, producing sub-micron-sized MOF_WA_ with exceptional water stability (Fig. 1A and B). To better adapt to the physiological conditions necessary for cell survival, the synthesis system was changed to saline (SA), and the resulting product was named MOF_SA_ (Fig. 1C and D). Elemental analysis confirmed the successful synthesis of MOF_WA_ and MOF_SA_ from the reaction between Zn^2+^ and ICA (Fig. S1). Scanning electron microscopy (SEM) and transmission electron microscopy (TEM) analyses were utilized to characterize the detailed morphology of the synthesized MOFs. MOF_WA_ was characterized by flaky base spirals that create hollow spheres with a diameter of approximately 500 nm (Fig. S2A). This hollow structure was attributed to Ostwald Ripening during the synthesis [44]. Meanwhile, in the saline synthesis system, MOF_SA_ demonstrated a morphology of solid spheres with a larger diameter of 2–3 μm, resulting from the self-assembly process during polymerization, which was driven by the increased surface tension due to the presence of sodium chloride. As evidence, unfused and semi-fused small nanoparticles ranging from 100 to 300 nm were observed surrounding the larger solid spheres under both TEM (Fig. S2B) and SEM (Fig. S3). Before introducing RBCs, the hemolysis rates of the key precursors, zinc ions (Zn^2+^) and imidazole-2-formaldehyde (ICA), within the system were assessed. The observed hemolysis rate consistently remained below 1 % (Fig. S4). These findings confirm the excellent hemocompatibility of the raw materials used in synthesizing the MOF armor. Thus, an in-situ coating method of the MOF layer onto the surface of natural RBCs was inspired by the assembly behavior during synthesis in saline. The saline system drives the attachment of MOF particles onto the surface of RBCs to reduce surface energy and minimize osmotic pressure fluctuations, thus helping to mitigate irreversible harmful effects on viable cells. MOF@RBC was produced by incorporating RBCs as one of the living synthetic components (Fig. 1E and F). This method represents a significant advancement through the integration of MOFs into living biological systems. In the in-situ coating strategy (MOF@RBC), a uniform protective shell of MOF, serving as a continuous nano-bio interface, was formed covering the intact surface of RBCs (Fig. 1F and Fig. S5A). In comparison to in-situ coating, a post-coating method was carried out by directly mixing the pre-synthesized MOF_SA_ with RBCs while stirring for 10 min to obtain MOF_SA_ + RBC (Fig. 1G and H). The post-coating method can only lead to a discontinuous coating of MOF_SA_ with partially exposed RBCs (Fig. 1H and Fig. S5B). To further evaluate the composite structures of MOF@RBC and MOF_SA_ + RBC, RBCs were removed by washing with pure water, leaving the pure MOF shell structures (Fig. S6). Both MOF@RBC and MOF_SA_ + RBC exhibited shell structures containing cavities. The shell structure of MOF@RBC forms a homogeneous whole (Fig. S6A), whereas the shell of MOF_SA_ + RBC comprises distinct small particles (Fig. S6B). TEM analysis at higher magnifications revealed that the MOF shell of both MOF@RBC and MOF_SA_ + RBC contains abundant hierarchical pore structures beyond the intrinsic nanopores of MOF, facilitating molecular exchange with the external environment (Fig. S7). To elucidate the formation process of MOF_SA_ shells, MOF_SA_ + RBC-S was synthesized by physically mixing MOF_SA_ and RBCs for 10 s. Sedimentation experiment at 4 °C for 12 h demonstrated that MOF_SA_ + RBC-S led to noticeable stratification between MOF_SA_ and RBCs. However, this stratification disappeared in MOF_SA_ + RBC, indicating an active interaction between MOF_SA_ and RBCs. At the same time, the sediment volume increased from MOF_SA_ + RBC-S and MOF_SA_ + RBC to MOF@RBC, attributed to the continuous formation of a nano-bio interface. This suggests that more water molecules entered the pores of the MOF shell, promoting the development of a porous structure capable of supporting RBCs (Fig. S8).

**Fig. 2:**
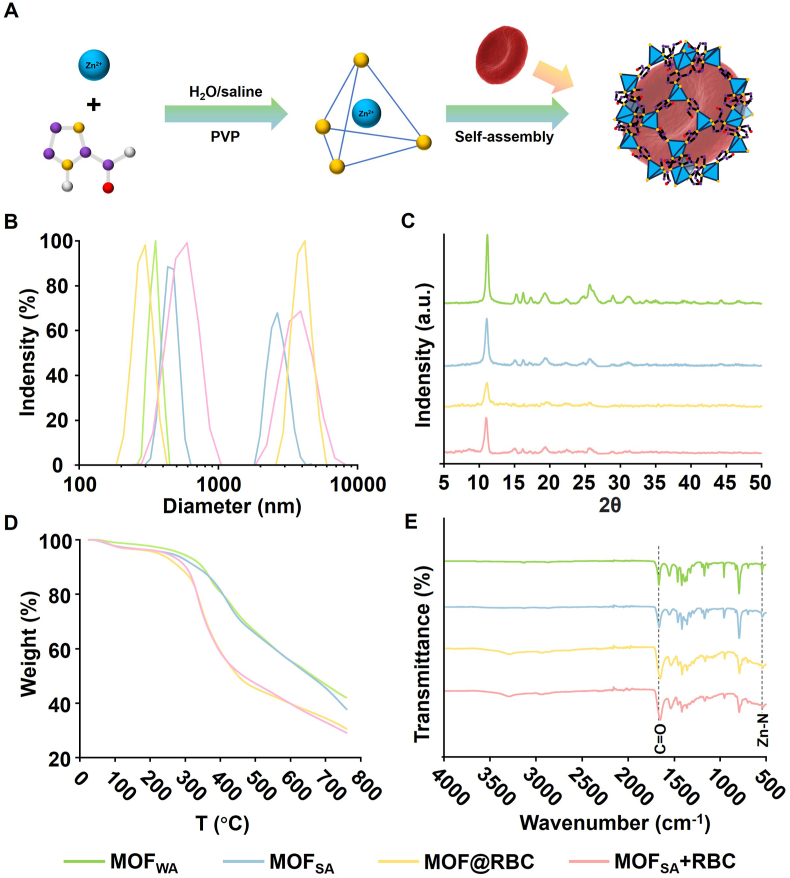
Characterization of the synthesized MOF-armored RBCs. (A) Conceptual formation of MOF-armored RBCs in a water or saline-based system, using zinc ions as the central ion and imidazole as an organic ligand. Carbon is purple, nitrogen is yellow, zinc is blue, oxygen is red, and hydrogen is white. (B) DLS, (C) XRD pattern, (D) TGA curve, and (E) FT-IR spectrum of MOF_WA_, MOF_SA_, MOF@RBC, and MOF_SA_ + RBC. (For interpretation of the references to colour in this figure legend, the reader is referred to the Web version of this article.)

The successful preparation of MOF-armored RBCs by Zn^2+^ and ICA reaction was further confirmed (Fig. 2) [45]. Energy-dispersive X-ray spectroscopy (EDS) elemental mappings determined the molecular formula of both MOF_WA_ and MOF_SA_ as [Zn(Imidazole-2-formaldehyde)]n (Table S1), indicating that zinc ions serve as key nodes holding the framework through coordination bonds with ICA (Fig. 2A). Dynamic light scattering (DLS) analysis was used to assess the hydrodynamic sizes and distribution of particles in solution (Fig. 2B). MOF_WA_ exhibited good dimensional homogeneity, with a hydrodynamic size of 670 ± 5 nm, aligning well with the SEM and TEM images. MOF_SA_ demonstrated a larger peak centered at 2.6 μm, confirming the formation of a solid spherical assembly (Fig. S3). Both MOF@RBC and MOF_SA_ + RBC displayed similar bimodal distributions, with the peak at 299 nm for MOF@RBC being smaller than the peak at 595 nm for MOF_SA_ + RBC, attributed to interference from RBCs in the self-assembly of free MOF nanoparticles. Their major peaks at around 3.8 μm correspond to the MOF/RBC composites, which is consistent with the electron microscope results. X-ray diffraction (XRD) analysis showed that the crystal structures of MOF_SA_, MOF@RBC, and MOF_SA_ + RBC are well-maintained and exhibit the same diffraction peaks as those of MOF_WA_, while RBCs do not show crystal structures (Fig. 2C and Fig. S9A). The thermal properties of the obtained MOFs were investigated using thermogravimetric analysis (TGA) (Fig. 2D) [40]. The initial weight loss around 100 °C can be attributed to the trapped water within the pores. The rapid weight loss above 300 °C is likely due to the deformation of the framework. MOF@RBC and MOF_SA_ + RBC exhibited comparable maximum decomposition temperatures at 339 °C, but a more notable weight loss rate, which can be attributed to the carbonization of the loaded RBCs (Fig. S9B). Fourier transform infrared spectroscopy (FTIR) was utilized to identify the presence of the associated enriched functional groups (Fig. 2E) [46]. A characteristic peak was observed at a wavenumber of 1665 cm^−1^, corresponding to the C=O bond of the aldehyde group in the ligand, which is distinct from the peak at 1648 cm^−1^ in RBCs (Fig. S9C). The band at 545 cm^−1^ indicates Zn-N stretching, confirming that the zinc ion coordinates with the nitrogen (N) in imidazole. Moreover, the FTIR spectra for both MOF@RBC and MOF_SA_ + RBC exhibited a shift in the characteristic peaks of C=O toward lower wavenumbers compared to MOF_WA_ and MOF_SA_, possibly due to interactions between the MOFs and RBCs.

**Fig. 3:**
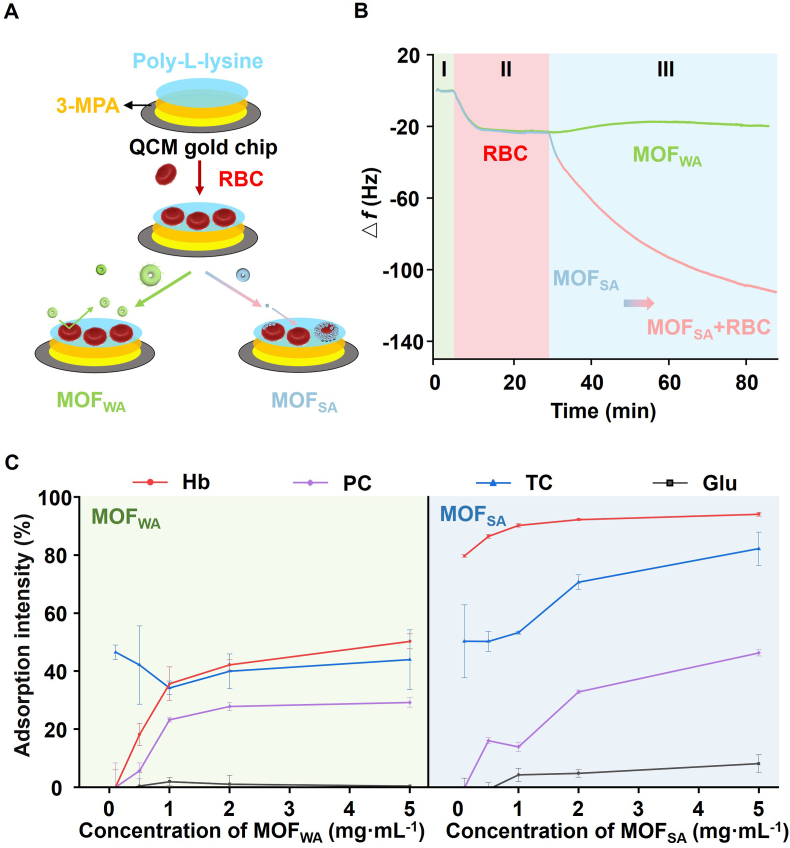
Determining the interactions between MOFs and RBCs. (A) Schematic of RBC-based QCM analysis for in situ detection of MOF-cell membrane interactions. RBC-QCM sensors were prepared by coating a gold piezoelectric sensor with 3-mercaptopropionic acid and poly-L-lysine, then immobilizing RBCs. Frequency response was monitored using MOF dispersion as the mobile phase. (B) Real-time changes in frequency (△ f) in response to the successive addition of RBCs and their subsequent adherence to MOF_SA_ indicate a substantial capture of MOF_SA_ NPs by RBCs. (C) Adsorption intensity of MOF_WA_ or MOF_SA_ for representative models of cell membrane components, including hemoglobin (Hb), glucose (Glu), total cholesterol (TC), and phosphatidylcholine (PC) (n = 3). (For interpretation of the references to colour in this figure legend, the reader is referred to the Web version of this article.)

The formation of spore-like structures on the surface of RBCs by MOFs is considered a crucial factor for cryopreservation. Thus, a quartz crystal microbalance with dissipation (QCM-D) biosensor was used to non-invasively investigate the real-time interaction kinetics between RBCs and MOFs (Fig. 3A). The poly-lysine coating created a gold chip that supports RBCs. Changes in the amount of mass adsorbed were measured through variations in frequency (△f). After establishing stable baselines in stage I, the flow was resumed by switching from saline to a suspension of RBCs. In stage II, a significant decrease in frequency (23 Hz) was observed, indicating that the RBCs underwent sedimentation and reached the electrode surface due to gravitational influence. Immediately afterward, these cells interacted with the poly-L-lysine on the electrode surface. The subsequent addition of MOF_WA_ or MOF_SA_ led to significantly different QCM-D shifts during stage III. For MOF_WA_, the frequency showed a slight increase, eventually reaching a state of dynamic equilibrium. In contrast, for MOF_SA_, the frequency response value was finally achieved at −110 Hz. Such a significant frequency change indicates a substantial capture of MOF_SA_ NPs by RBCs and strong interactions between them (Fig. 3B). Moreover, optical microscopy revealed clear binding of MOF_SA_ NPs to RBCs, whereas no such interaction was observed between MOF_WA_ NPs and RBCs (Supplementary Movie S1). It was suggested that the interaction behavior between MOF_SA_ and RBCs differs from that observed between MOF_WA_ and RBCs, and MOF_SA_ synthesized in saline can autonomously adsorb onto the surface of RBCs, forming a protective shell.

The surface zeta potential for MOF NPs was measured. MOF_SA_ NPs exhibited a negative charge, indicating that the interactions between the MOF_SA_ and the RBC surface were not due to strong electrostatic forces (Fig. S10). Given the complexity of the components on the cell membrane, four different substances, *i.e.*, hemoglobin (Hb), glucose (Glu), total cholesterol (TC), and phosphatidylcholine (PC), were selected as representative models to study the binding sites of MOFs (Fig. 3C). Specifically, the adsorption percentages of MOF_WA_ for these four substances were all below 50 %, even at a high MOF_WA_ concentration of 5 mg mL^−1^. In contrast, MOF_SA_ exhibited strong adsorption for Hb, reaching 80 % at a low MOF_SA_ concentration of 0.1 mg mL^−1^. Additionally, MOF_SA_ showed a relatively high adsorption intensity for TC. In summary, the interaction between MOF_SA_ and RBCs is a highly specific recognition process. MOF_SA_ selectively identifies counterpart proteins and cholesterol on the RBC membrane, forming a protective shell around the cells.

### 3.2 Cryopreservation of RBCs mediated by MOF armor

The cryoprotective effectiveness of armor structures was evaluated through a freeze-thawing cycle to demonstrate the potential of synthesized MOF armor in enhancing the cryopreservation of sheep RBCs. In the unique structure of the MOF@RBC and MOF_SA_ + RBC, RBCs were protected by self-assembled MOF armor. Thus, to determine cell recovery post-freeze-thawing, the protective MOF shell was removed by gradually adding a citrate buffer (pH 6.25), matching saline osmolality (Table S2), and achieving over 85 % MOF degradation (Table S3) [47]. Hemolysis assay showed that the citric acid isotonic degradation solution itself does not cause cell damage (Fig. S11). Ultrasonic treatment after MOF degradation resulted in 0 % cell recovery for MOF_SA_ + RBC-S, MOF_SA_ + RBC, and MOF@RBC, confirming complete RBC detachment from the MOF shell and eliminating false positives (Fig. S12). The cryopreservation performance of MOF_SA_ + RBC and MOF@RBC, loaded with varying RBC quantities, was evaluated (Fig. S13). No significant difference in survival rate was observed for MOF_SA_ + RBC at RBC counts of 1 and 5 × 10^8^, likely because MOF_SA_ particles had already self-assembled, preventing excessive RBC armor formation. MOF@RBC showed better survival at 5 × 10^8^ RBCs, which was therefore chosen for cryopreservation. At 1 × 10^8^ RBCs, MOF@RBC's armor was relatively thick, increasing the risk of unloading damage. Conversely, at 9 × 10^8^ RBCs, MOF_SA_ might be insufficient to form a continuous protective layer. The biocompatibility of MOF armor was then investigated. MOF_WA_ and MOF_SA_ + RBC-S showed hemolysis percentages below 8 %, while MOF_SA_ + RBC exhibited a hemolysis rate of 25 %. The hemolysis rate of MOF@RBC was slightly lower than that of MOF_SA_ + RBC, at 18 % (Fig. S14). This reduction may be due to the initial synthesis involving RBCs, which helps reduce mechanical damage from collisions between larger MOF_SA_ particles and RBCs. While encapsulation and controlled release can cause some cellular damage, the significantly improved protective effectiveness of MOF_SA_ armor against extensive ice crystal formation during freezing cycles is a major advantage. Future efforts to optimize the spore-like microstructure of MOF-armed RBCs are expected to reduce armor-processing damage and improve biocompatibility. Additionally, MTS analysis showed that the MOF@RBC did not show cytotoxicity to NIH/3T3 cells when the doses of MOF_SA_ ranged from 0 to 50 μg MOF_SA_/10^6^ cells (Fig. S15).

**Fig. 4:**
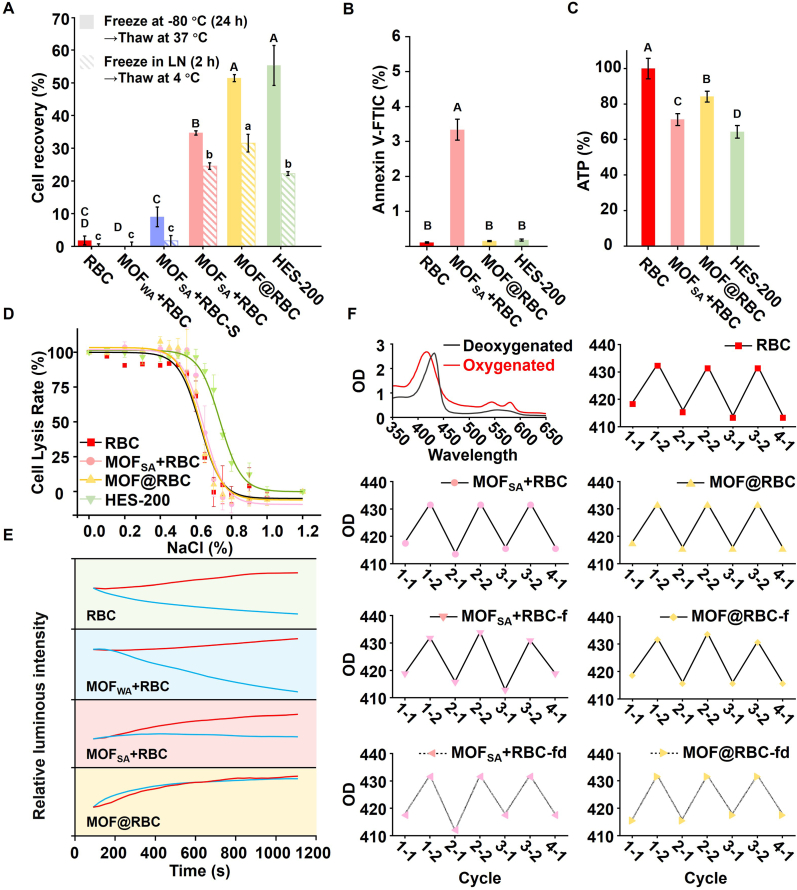
Assessment of MOF-based materials for cryopreservation. (A) Recovery rates of sheep RBCs cryopreserved with MOF structures or HES polymer (200 mg mL^−1^) saline solutions. MOF_WA_ + RBC was created by mixing pre-synthesized MOF_WA_ and RBCs for 10 min; MOF_SA_ + RBC-S by mixing pre-synthesized MOF_SA_ and RBCs for 10 s (distinct from the 10-min MOF_SA_ + RBC protocol). (B) Membrane phosphatidylserine exposure, (C) ATP levels, and (D) osmotic shock resistance of recovered RBCs compared to native RBCs. (E) Oxygenation curves over time for native and MOF-armored RBCs after adding luminol-perborate mixture (red lines: pre-freeze-thaw; blue lines: post-freeze-thaw). (F) UV–vis spectra of MOF-armored RBCs in oxygenated and deoxygenated states. Here, -f indicates the sample has undergone freeze-thawing, while -fd indicates that it has been treated for freeze-thawing followed by degradation of MOFs. *p* < 0.05. (For interpretation of the references to colour in this figure legend, the reader is referred to the Web version of this article.)

Both the mild condition (frozen at −80 °C for 24 h, and thawed at 37 °C.) and the strict condition (rapid immersion in liquid nitrogen for 2 h, then slowly thawed at 4 °C) were performed. Remarkably, MOF@RBC achieved the highest cell recovery of 51 % at a concentration of 1 mg mL^−1^ under mild test conditions, matching the performance of the commercial cryoprotectant HES, which requires a much higher concentration of 200 mg mL^−1^ to achieve the same effect. Even under stringent freeze-thawing conditions, MOF@RBC demonstrated superior preservation, with a 32 % recovery rate, outperforming HES and highlighting the exceptional cryoprotective potential of this MOF-based one-step loading strategy (Fig. 4A). In comparison, MOF_WA_ failed to protect in mild freeze-thawing conditions, while the incorporation of MOF_SA_ improved the recovery rate from 9 % to 35 % with extended mixing time (from MOF_SA_ + RBC-S to MOF_SA_ + RBC), which can be attributed to the formation of MOF_SA_ armor. SEM analysis further confirmed the structural integrity of MOF@RBC and MOF_SA_ + RBC after freeze-thawing, whereas MOF_SA_ + RBC-S failed to form the armored RBC structure (Fig. S16). This indicates that forming spore-like structures in MOF-armored RBCs is essential for effective cryopreservation. Furthermore, ICP analysis revealed that the zinc ions leached from MOF@RBC after freeze-thaw treatment were only 2.08 ± 0.08 %, providing strong evidence of the MOF armor's stability during the freeze-thaw process. A single wash after the degradation of the MOF armor was enough to reduce the zinc ion concentration to as low as 1.09 ± 0.03 % (Fig. S17). Notably, the zeta potential of RBCs rose from −11.80 ± 1.22 mV to −9.26 ± 2.68 mV after MOF shell adsorption and returned to −12.02 ± 0.99 mV following freeze-thaw and MOF degradation. The RBC size distribution showed no significant difference in both MOF_SA_ + RBC-fd and MOF@RBC-fd (Fig. S18). SEM images showed that the RBCs in MOF@RBC and MOF_SA_ + RBC maintained their original biconcave disc shapes, demonstrating excellent structural preservation (Fig. S19). Optical microscopy further confirmed the presence of intact RBCs following freeze-thawing under the protection of the MOF shell (Fig. S20). Collectively, these results highlight MOF@RBC as an effective cryoprotectant, offering superior preservation compared to traditional HES while requiring significantly lower concentrations. Furthermore, flow cytometry experiments showed that the exposure of phosphatidylserine (PS) on the outer membrane of RBCs after the freeze-thaw process, as well as the degradation of MOF@RBC, remained unchanged, as determined by an Annexin V-binding assay kit (Fig. 4B and Fig. S21) [48]. Although the ATP levels in RBCs cryopreserved with MOF@RBC are lower than those in fresh RBCs (Fig. 4C), they still performed better than groups with MOF_SA_ + RBC and those without HES. The deformability of RBCs was not affected, as measured by the microporous filtration method (Fig. S22). Additionally, compared to fresh RBCs, RBCs cryopreserved with MOF_SA_ armor in both MOF_SA_ + RBC and MOF@RBC showed the same osmotic shock resistance, outperforming those preserved with HES (Fig. 4D).

A luminol-based chemiluminescence measurement was used to evaluate hemoglobin-related behavior in RBCs (Fig. 4E). After adding luminol-perborate to saline, native RBCs, MOF_WA_, armored MOF@RBC, and MOF_SA_ + RBC exhibited chemiluminescence, increasing intensity over 20 min. This suggests that the armored shell did not hinder the entry of small molecules like luminol and peroxide into the RBC due to the inherent porosity of MOFs. Notably, the luminescence intensity of both unprotected RBCs and MOF_WA_ + RBC diminished after freeze-thawing, suggesting that cryoinjury impairs the iron catalytic properties of intracellular hemoglobin within RBCs. In MOF_SA_ + RBC, binding RBCs to MOF_SA_ alleviated the decline in luminous intensity due to freezing. Remarkably, the MOF@RBC maintained an increased luminous intensity even after freeze-thawing cycles, demonstrating the effectiveness of the MOF armor in reducing cryodamage. The oxygen-carrying capacity was a crucial feature of RBCs (Fig. S23). UV–vis spectroscopy demonstrated the reversible shift of maximum absorption peaks of RBCs in both oxygenated and deoxygenated states (Fig. 4F). The characteristic absorption peak for both natural and armored RBCs in the oxygenated state was 416 nm. After nitrogen bubbling and adding Na_2_S_2_O_4_, this peak red-shifted to 432 nm. Even after freeze-thawing, the oxygen binding and release processes remained repeatable, indicating that the oxygen-carrying capacity of the RBC was well-preserved before and after MOF degradation (Fig. 4F).

### 3.3 MOF-mediated mechanisms for inhibiting ice-induced damage

**Fig. 5:**
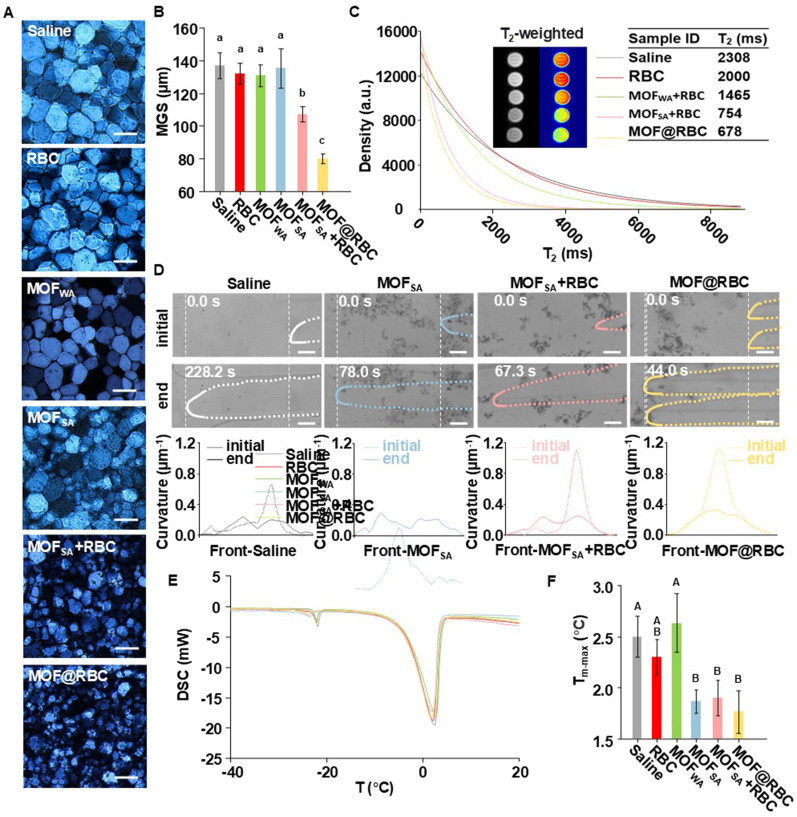
Investigation into the ice inhibition mechanism of MOF-armored RBCs. (A) Images of ice crystals grown in saline with various MOF structures, annealed at −9 °C for 30 min. The scale bar is 100 μm. (B) Quantitative analysis of ice crystal maximum grain size (MGS). N = 3. (C) T_2_ inversion spectra measured at 35 °C using a low-field NMR spectrometer; inset shows T_2_-weighted images at 32 °C (brighter signals indicate longer T_2_ values). The inserted table lists T_2_ peak values calculated via the Carr-Purcell-Meiboom-Gill (CPMG) sequence. (D) DIGM images showing the effects of MOF_SA_, MOF_SA_ + RBC, and MOF@RBC on ice front curvature during directional solidification in saline (images taken at 300 μm intervals). The bottom plot displays ice front curvature at 0.0 s (dashed line) and end times (Saline: 228.2 s; MOFSA: 78.0 s; MOFSA + RBC: 67.3 s; MOF@RBC: 44.0 s, solid line). The scale bar is 50 μm. (E) Typical DSC melting curves of frozen dispersion droplets (10 mg) heated at 2 °C·min^−1^. (F) Melting temperatures (T_m-max_) corresponding to the highest peak of frozen droplets with various samples. *p* < 0.05.

To better understand MOF-assisted cryopreservation, a “splat” assay was utilized to quantitatively evaluate the ice recrystallization inhibition (IRI) activity. The reduction in ice crystal size indicates an increase in IRI activity [49]. The polarized optical microscopy image of recrystallized ice crystals after the addition of MOF@RBC exhibited the smallest grain size in comparison to the negative control (saline) (Fig. 5A and Fig. S24). The quantitative analysis estimated the maximum grain size (MGS) in MOF@RBC to be 58 % relative to saline, with MOF_SA_ + RBC following at 78 % (Fig. 5B). These findings demonstrate that MOF-armored RBCs effectively inhibit ice crystal recrystallization. The higher IRI activity of MOF@RBC compared to MOF_SA_ + RBC can be attributed to the former's more uniform MOF protective shell. In contrast, neither MOF_WA_ nor MOF_SA_ showed any measurable IRI activity in the absence of RBCs. Similarly, both before and after freeze-thawing, RBCs alone were negative for IRI activity. (Fig. 5B and Fig. S25A). To confirm the critical role of the self-assembled spore-like structure in inhibiting ice recrystallization, MOF_WA_ + RBCs and MOF_SA_ + RBC-S were prepared, revealing that non-spore-like structures lack IRI activity (Fig. S25). Thus, one possible reason MOF-armored RBCs improve the recovery of cryopreserved RBCs is their ability to inhibit ice recrystallization during thawing, which is a major factor in cell death [5].

To explore the mechanism underlying the interaction between MOF-armored RBCs and ice at the molecular level, the NMR proton spin-spin relaxation time T_2_ was measured. This parameter, highly sensitive to water mobility, investigates the impact of MOF-armored RBCs on the dynamics of water molecules within the system. Although the test temperature here is not the actual freezing temperature, it is reasonable to predict that the relative ability of the material to bind liquid water molecules will remain unchanged. Both T_2_ inversion spectra and T_2_-weighted images were acquired (Fig. 5C) [50]. Notably, the images of MOF@RBC and MOF_SA_ + RBC displayed significantly darker signal intensity compared to those of native RBCs and MOF_WA_, and the formation of spore-like structures of MOF-armored RBCs decreased the T_2_ of the RBC solution, suggesting that water mobility was hindered, possibly due to the binding of water molecules by the pores of the MOF shell. Furthermore, an increase in T_2_ values was observed across the samples in the following order: MOF@RBC < MOF_SA_ + RBC < MOF_WA_ < RBC < Saline, aligning with the trend observed via MGS. ATR-FTIR analysis revealed a red shift in the OH stretching vibration peaks for MOF@RBC and MOF_SA_ + RBCs (3290 cm^−1^) compared to MOF_SA_ (3331 cm^−1^), suggesting enhanced hydrogen bonding between the MOF armor and water molecules (Fig. S26). This binding of the MOF shell to water molecules increased the energetic barrier for water incorporation into the ice lattice [51]. Consequently, this interaction slowed down the process of ice recrystallization.

In addition to IRI, the morphology of ice crystals also affects the efficiency of cell cryopreservation [16]. Thus, a homemade cooling pool was employed to investigate this further. It was maintained on one side at −30 °C and the other at ambient conditions at 15 °C, thereby establishing a spatial temperature gradient spanning 2.5 cm. Saline solution confined within the microfluidic channel undergoes gradual growth of crystalline ice from cold to ambient areas, visualized through directional ice growth microscopy (DIGM) images (Fig. 5D, right to left). The transition of the ice front morphology from sharp at the initial time point to smooth after 300 μm was carefully recorded, with time intervals measured to determine critical transition velocities. Meanwhile, the evolution of curvature was monitored, and the values at both the initial and final time points were recorded, where the maximum curvature peak significantly disappeared at the endpoint. In the control saline group, the presence of ions in the matrix caused the ice-water interface to exhibit a sawtooth-like morphology during rapid growth rates at 15 μm/s (Fig. S27) [52]. As growth slowed, this morphology transitioned to smoother fronts, resulting in a critical velocity of 2.0 ± 0.9 μm/s. The addition of MOF_SA_, MOF_SA_ + RBC, and MOF@RBC particles increased the critical velocities for ice front transitions to 4.0 ± 0.3, 4.7 ± 0.4, and 6.6 ± 0.3 μm/s, respectively, promoting curvature transformations at the ice growth front. For RBC cryopreservation, smooth ice crystal morphologies are preferable to spiculated ones, as they reduce the likelihood of cellular membrane damage during freezing storage caused by sharp ice protrusions [53]. Thus, the strong ability of MOF@RBC to smooth ice crystals makes it a promising candidate for enhancing RBC cryopreservation outcomes by minimizing cryodamage. Additionally, the growth of a single ice crystal during freezing was determined (Supplementary Movie S2). The ice crystals formed flat disk shapes in the presence of both MOF@RBC and MOF_SA_. This finding suggested that MOF_SA_ NPs did not interact with specific planes of the ice crystals during freezing, thereby maintaining the ice crystals' smooth morphology [54]. Notably, at the same temperature of −0.2 °C, MOF@RBC exhibited a slower growth rate of ice crystals than MOF_SA_, confirming its role in inhibiting ice crystal growth (Fig. S28).

**Fig. 6:**
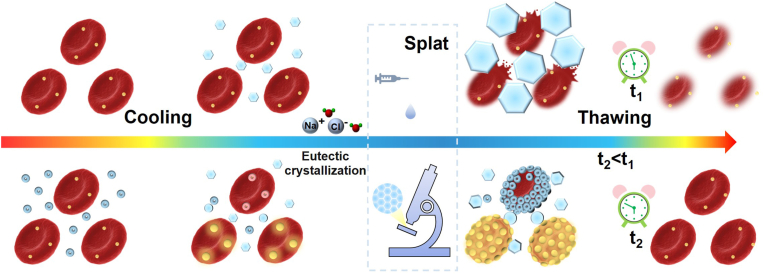
Proposed cryopreservation mechanisms of MOF-armored RBC. The proposed mechanisms include 1) decreasing ice grain size by reducing ice recrystallization; 2) smoothing the ice growth front through enhanced thermal conductivity; 3) suppressing NaCl eutectic formation; and 4) minimizing cellular exposure duration to the ice phase by accelerating ice melting.

Differences in thermal conductivity among solid, liquid, and particle materials affect the solid-liquid interface shape [55]. Thus, the impact of incorporating MOF-armored RBCs on the thermal conductivity of the system was examined using differential scanning calorimetry (DSC). It was demonstrated that frozen dispersions of MOF_SA_, MOF_SA_ + RBC, and MOF@RBC melted sooner than both saline and MOF_WA_ dispersions (Fig. 5E and F). The observation that melting was facilitated indicated that MOF_SA_, MOF_SA_ + RBC, and MOF@RBC enhance thermal conductivity, supporting the experimental findings that they facilitate the smoothing of the ice growth front (Fig. 5D). Unlike IRI, this property is inherent to MOF_SA_ NPs and does not require the formation of spore-like structures. In cryopreservation, the longer cells are exposed to the ice phase during melting, the less favorable it becomes for maintaining cell viability. Therefore, the effectiveness of MOF_SA_ in accelerating ice melting is another crucial factor in enhancing cryopreservation. Additionally, eutectic crystallization may be one of the mechanisms causing damage during cell cryopreservation [56]. DSC analyses revealed that MOF_SA_, MOF_SA_ + RBC, and MOF@RBC significantly suppressed the eutectic formation of NaCl at −22 °C, potentially contributing to improved cryopreservation recovery of RBCs. Based on the analysis above, MOF-armored RBCs are highly promising candidates as cryoprotectants due to their multiple abilities to inhibit ice growth, smooth the shape of the ice front, promote melting, and reduce eutectic formation (Fig. 6).

## 4 Conclusion

In summary, Zn-based MOFs that can actively form RBC protective shells were successfully constructed under physiological conditions. The formation of spore-like MOF-armored RBCs was demonstrated for the first time, showcasing exceptional ice-control properties, including inhibiting ice growth and recrystallization, smoothing the shape of the ice front, accelerating ice melting, and suppressing eutectic crystallization. Compared to MOF_SA_ + RBC, MOF@RBC employed a one-step in situ loading method, demonstrating a more uniform MOF shell, which significantly improved sheep RBC recovery after freeze-thawing and established it as a promising cryoprotectant. The ability of MOF armor to inhibit ice crystal growth likely stems from its porous structure, which restricts the movement of water molecules through hydrogen bonding. Additionally, MOF_SA_ serves as a “catalyst” by suppressing sodium chloride eutectic formation and accelerating ice melting. Despite current studies on MOF armored-RBC cryopreservation, future work should focus on validating human RBC cryopreservation and assessing immunogenicity to support clinical translation. This advancement paves the way for the design and development of novel MOF-inspired architectures for cryopreservation, broadening the versatility of the armor strategy to a wide range of biological applications.

## CRediT authorship contribution statement

**Xin Li:** Writing – original draft, Visualization, Validation, Methodology, Formal analysis, Data curation. **Zhaoyang Gong:** Validation, Investigation. **Muzhe Xu:** Validation, Methodology. **Song Wang:** Writing – review & editing, Formal analysis. **Bingbing Sun:** Writing – review & editing, Resources, Methodology, Funding acquisition, Formal analysis, Data curation, Conceptualization.

## Declaration of competing interest

The authors declare that they have no known competing financial interests or personal relationships that could have appeared to influence the work reported in this paper.

## Data Availability

Data will be made available on request.

## Appendix A Supplementary data

Additional characterization data for nanoparticles include SEM and TEM analysis, ice recrystallization inhibition activity assays, ice front growth morphology and curvature, and ATR-FTIR analysis.

## References

[1] Carson J.L., Stanworth S.J., Guyatt G., Valentine S., Dennis J., Bakhtary S., Cohn C.S., Dubon A., Grossman B.J., Gupta G.K., Hess A.S., Jacobson J.L., Kaplan L.J., Lin Y., Metcalf R.A., Murphy C.H., Pavenski K., Prochaska M.T., Raval J.S., Salazar E., Saifee N.H., Tobian A.A.R., So-Osman C., Waters J., Wood E.M., Zantek N.D., Pagano M.B. (2023). Red blood cell transfusion: 2023 AABB international guidelines. JAMA.

[2] Villa C.H., Anselmo A.C., Mitragotri S., Muzykantov V. (2016). Red blood cells: supercarriers for drugs, biologicals, and nanoparticles and inspiration for advanced delivery systems. Adv. Drug Deliv. Rev..

[3] Scott K.L., Lecak J., Acker J.P. (2005). Biopreservation of red blood cells: past, present, and future. Transfus. Med. Rev..

[4] Whaley D., Damyar K., Witek R.P., Mendoza A., Alexander M., Lakey J.R. (2021). Cryopreservation: an overview of principles and cell-specific considerations. Cell Transplant..

[5] Fowler A., Toner M. (2006). Cryo‐injury and biopreservation. Ann. N. Y. Acad. Sci..

[6] He Z., Liu K., Wang J. (2018). Bioinspired materials for controlling ice nucleation, growth, and recrystallization. Acc. Chem. Res..

[7] Chang T., Zhao G. (2021). Ice inhibition for cryopreservation: materials, strategies, and challenges. Adv. Sci..

[8] Ma Y., Gao L., Tian Y., Chen P., Yang J., Zhang L. (2021). Advanced biomaterials in cell preservation: hypothermic preservation and cryopreservation. Acta Biomater..

[9] Best B.P. (2015). Cryoprotectant toxicity: facts, issues, and questions. Rejuvenation Res..

[10] Pallotta V., D'Amici G.M., D'Alessandro A., Rossetti R., Zolla L. (2012). Red blood cell processing for cryopreservation: from fresh blood to deglycerolization, blood cells, molecules. Diseases.

[11] Voets I.K. (2017). From ice-binding proteins to bio-inspired antifreeze materials. Soft Matter.

[12] Marshall C.B., Fletcher G.L., Davies P.L. (2004). Hyperactive antifreeze protein in a fish. Nature.

[13] Mitchell D.E., Lovett J.R., Armes S.P., Gibson M.I. (2016). Combining biomimetic block copolymer worms with an ice-inhibiting polymer for the solvent-free cryopreservation of red blood cells. Angew. Chem. Int. Ed..

[14] Eickhoff L., Keßler M., Stubbs C., Derksen J., Viefhues M., Anselmetti D., Gibson M.I., Hoge B., Koop T. (2023). Ice nucleation in aqueous solutions of short- and long-chain poly(vinyl alcohol) studied with a droplet microfluidics setup. J. Chem. Phys..

[15] Georgiou P.G., Kinney N.L.H., Kontopoulou I., Baker A.N., Hindmarsh S.A., Bissoyi A., Congdon T.R., Whale T.F., Gibson M.I. (2022). Poly(vinyl alcohol) molecular bottlebrushes nucleate ice. Biomacromolecules.

[16] Yang J., Liu M., Zhang T., Ma J., Ma Y., Tian S., Li R., Han Y., Zhang L. (2022). Cell‐friendly regulation of ice crystals by antifreeze organism‐inspired materials. AIChE J..

[17] Ma Y., Zhang J., Tian Y., Fu Y., Tian S., Li Q., Yang J., Zhang L. (2023). Zwitterionic microgel preservation platform for circulating tumor cells in whole blood specimen. Nat. Commun..

[18] Geng H., Liu X., Shi G., Bai G., Ma J., Chen J., Wu Z., Song Y., Fang H., Wang J. (2017). Graphene oxide restricts growth and recrystallization of ice crystals. Angew. Chem. Int. Ed..

[19] Bai G., Song Z., Geng H., Gao D., Liu K., Wu S., Rao W., Guo L., Wang J. (2017). Oxidized quasi-carbon nitride quantum dots inhibit ice growth. Adv. Mater..

[20] (2012). Introduction to metal–organic frameworks. Chem. Rev..

[21] Ali Akbar Razavi S., Morsali A. (2019). Linker functionalized metal-organic frameworks. Coord. Chem. Rev..

[22] Furukawa H., Cordova K.E., O'Keeffe M., Yaghi O.M. (2013). The chemistry and applications of metal-organic frameworks. Science.

[23] Stock N., Biswas S. (2012). Synthesis of metal-organic frameworks (MOFs): routes to various MOF topologies, morphologies, and composites. Chem. Rev..

[24] Wang B., Côté A.P., Furukawa H., O'Keeffe M., Yaghi O.M. (2008). Colossal cages in zeolitic imidazolate frameworks as selective carbon dioxide reservoirs. Nature.

[25] N. Zheng, Q. Wang, Z. Cao, C. Yu, R. Zhang, K. Xiang, H. Wu, K. Li, Q. Ni, Q. Ma, J. Mu, X. Chen, L. He, S. Liu, Multifunctional H2S-activated metal-organic framework systems for targeted colorectal cancer imaging and synergistic copper-induced tumor regression, BMEMat n/a (n.d.) e70029. 10.1002/bmm2.70029.

[26] Lee D., Lee S., Choi I., Kim M. (2024). Positional functionalizations of metal–organic frameworks through invasive ligand exchange and additory MOF-on-MOF strategies: a review. Smart Molecules.

[27] Belmabkhout Y., Bhatt P.M., Adil K., Pillai R.S., Cadiau A., Shkurenko A., Maurin G., Liu G., Koros W.J., Eddaoudi M. (2018). Natural gas upgrading using a fluorinated MOF with tuned H2S and CO2 adsorption selectivity. Nat. Energy.

[28] Rogge S.M.J., Bavykina A., Hajek J., Garcia H., Olivos-Suarez A.I., Sepúlveda-Escribano A., Vimont A., Clet G., Bazin P., Kapteijn F., Daturi M., Ramos-Fernandez E.V., i Xamena F.X.L., Speybroeck V.V., Gascon J. (2017). Metal–organic and covalent organic frameworks as single-site catalysts. Chem. Soc. Rev..

[29] Li R., Guo W., Zhu Z., Zhai Y., Wang G., Liu Z., Jiao L., Zhu C., Lu X. (2023). Single-atom indium boosts electrochemical dopamine sensing. Anal. Chem..

[30] Zhang X., Yan M., Chen P., Li J., Li Y., Li H., Liu X., Chen Z., Yang H., Wang S., Wang J., Tang Z., Huang Q., Lei J., Hayat T., Liu Z., Mao L., Duan T., Wang X. (2025). Emerging MOFs, COFs, and their derivatives for energy and environmental applications. Innovation.

[31] Lawson H.D., Walton S.P., Chan C. (2021). Metal–organic frameworks for drug delivery: a design perspective. ACS Appl. Mater. Interfaces.

[32] Tran V.A., Thuan Le V., Doan V.D., Vo G.N.L. (2023). Utilization of functionalized metal–organic framework nanoparticle as targeted drug delivery system for cancer therapy. Pharmaceutics.

[33] Doonan C., Riccò R., Liang K., Bradshaw D., Falcaro P. (2017). Metal–organic frameworks at the biointerface: synthetic strategies and applications. Acc. Chem. Res..

[34] Zhu W., Guo J., Agola J.O., Croissant J.G., Wang Z., Shang J., Coker E., Motevalli B., Zimpel A., Wuttke S., Brinker C.J. (2019). Metal–organic framework nanoparticle-assisted cryopreservation of red blood cells. J. Am. Chem. Soc..

[35] Lei Q., Sun Y., Huang J., Liu W., Zhan X., Yin W., Guo S., Sinelshchikova A., Brinker C.J., He Z., Guo J., Wuttke S., Zhu W. (2023). Dimensional reduction of metal–organic frameworks for enhanced cryopreservation of red blood cells. Angew. Chem. Int. Ed..

[36] Guo J., Yu Y., Zhu W., Serda R.E., Franco S., Wang L., Lei Q., Agola J.O., Noureddine A., Ploetz E., Wuttke S., Brinker C.J. (2021). Modular assembly of red blood cell superstructures from metal–organic framework nanoparticle-based building blocks. Adv. Funct. Mater..

[37] Youn W., Kim J.Y., Park J., Kim N., Choi H., Cho H., Choi I.S. (2020). Single-cell nanoencapsulation: from passive to active shells. Adv. Mater..

[38] Zhu W., Guo J., Amini S., Ju Y., Agola J.O., Zimpel A., Shang J., Noureddine A., Caruso F., Wuttke S., Croissant J.G., Brinker C.J. (2019). SupraCells: living mammalian cells protected within functional modular nanoparticle-based exoskeletons. Adv. Mater..

[39] Cai P., Zhang X., Wang M., Wu Y.-L., Chen X. (2018). Combinatorial nano–bio interfaces. ACS Nano.

[40] Shieh F.-K., Wang S.-C., Leo S.-Y., Wu K.C.-W. (2013). Water-based synthesis of zeolitic imidazolate Framework-90 (ZIF-90) with a controllable particle size. Chem. Eur J..

[41] Zhang S., Bai H., Yang P. (2015). Real-time monitoring of mechanical changes during dynamic adhesion of erythrocytes to endothelial cells by QCM-D. Chem. Commun..

[42] Quickenden T.I., Cooper P.D. (2001). Increasing the specificity of the forensic luminol test for blood. Luminescence.

[43] Okamoto Y., Sugisaki S., Suga K., Umakoshi H. (2017). Development of time-course oxygen binding analysis for hemoglobin-based oxygen carriers. Anal. Sci..

[44] Huo J., Wang L., Irran E., Yu H., Gao J., Fan D., Li B., Wang J., Ding W., Amin A.M., Li C., Ma L. (2010). Hollow ferrocenyl coordination polymer microspheres with micropores in shells prepared by ostwald ripening. Angew. Chem. Int. Ed..

[45] Yang X., Tang Q., Jiang Y., Zhang M., Wang M., Mao L. (2019). Nanoscale ATP-responsive zeolitic imidazole Framework-90 as a general platform for cytosolic protein delivery and genome editing. J. Am. Chem. Soc..

[46] Fan C., Tang Y., Wang H., Huang Y., Xu F., Yang Y., Huang Y., Rong W., Lin Y. (2022). ZIF-90 with biomimetic Zn–N coordination structures as an effective nanozyme to mimic natural hydrolase. Nanoscale.

[47] Feng Y., Wang H., Zhang S., Zhao Y., Gao J., Zheng Y., Zhao P., Zhang Z., Zaworotko M.J., Cheng P., Ma S., Chen Y. (2019). Antibodies@MOFs: an in vitro protective coating for preparation and storage of biopharmaceuticals. Adv. Mater..

[48] Yasin Z., Witting S., Palascak M.B., Joiner C.H., Rucknagel D.L., Franco R.S. (2003). Phosphatidylserine externalization in sickle red blood cells: associations with cell age, density, and hemoglobin F. Blood.

[49] Biggs C.I., Stubbs C., Graham B., Fayter A.E.R., Hasan M., Gibson M.I. (2019). Mimicking the ice recrystallization activity of biological antifreezes. When is a new polymer “active”. Macromol. Biosci..

[50] Abdel-Aty H., Simonetti O., Friedrich M.G. (2007). T2-weighted cardiovascular magnetic resonance imaging. J. Magn. Reson. Imag..

[51] Bai G., Hu J., Qin S., Qi Z., Zhuang H., Sun F., Lu Y., Jin S., Gao D., Wang J. (2022). Small-molecule fulvic acid with strong hydration ability for non-vitreous cellular cryopreservation. iScience.

[52] Applied Surface Thermodynamics, (n.d.).

[53] Wang J.-H. (2000). A comprehensive evaluation of the effects and mechanisms of antifreeze proteins during low-temperature preservation. Cryobiology.

[54] Griffith M., Yaish M.W.F. (2004). Antifreeze proteins in overwintering plants: a tale of two activities. Trends Plant Sci..

[55] Li C., Luo Z., Qing S., Zhang J., Zhu J. (2025). Microscopic analysis of the influence of nanoparticle shape and solid-liquid interfacial layer density on the thermal conductivity of nanofluids: a molecular dynamics study on Cu-H2O nanofluids. Int. J. Therm. Sci..

[56] Han B., Bischof J.C. (2004). Direct cell injury associated with eutectic crystallization during freezing. Cryobiology.

