# Supplementary material for: Red blood cell-activated antifreeze metal-organic framework armor for cryopreservation
Source: Mater Today Bio. 2025 Nov 7;35:102529. doi: 10.1016/j.mtbio.2025.102529 (PMC12663683; doi:10.1016/j.mtbio.2025.102529)
Supplement: Multimedia component 1 [file mmc1.docx]

Supplementary Information

RBC-Activated Antifreeze Metal-Organic Framework Armor for Cryopreservation

Xin Li^[1,2,3]^, Zhaoyang Gong^[1,2,3]^, Muzhe Xu^[1,2,3]^, Song Wang^[4]^ and Bingbing Sun*^[1,2,3]^

**Affiliations:**

^1^ State Key Laboratory of Fine Chemicals, Dalian University of Technology, 2 Linggong Road, 116024 Dalian, China.

^2^ School of Chemical Engineering, Dalian University of Technology, 2 Linggong Road, 116024 Dalian, China.

^3^ Frontiers Science Center for Smart Materials Oriented Chemical Engineering, School of Chemical Engineering, Dalian University of Technology, 2 Linggong Road, 116024 Dalian, China.

^4^ State Key Laboratory of Mesoscience and Engineering, Institute of Process Engineering, Chinese Academy of Sciences, Beijing 100190, China

^*^Corresponding Authors. E-mail: bingbingsun@dlut.edu.cn

KEYWORDS: Ice recrystallization inhibition, armored structure, nano-bio interface, metal-organic frameworks, cryopreservation

Supporting figures and tables.


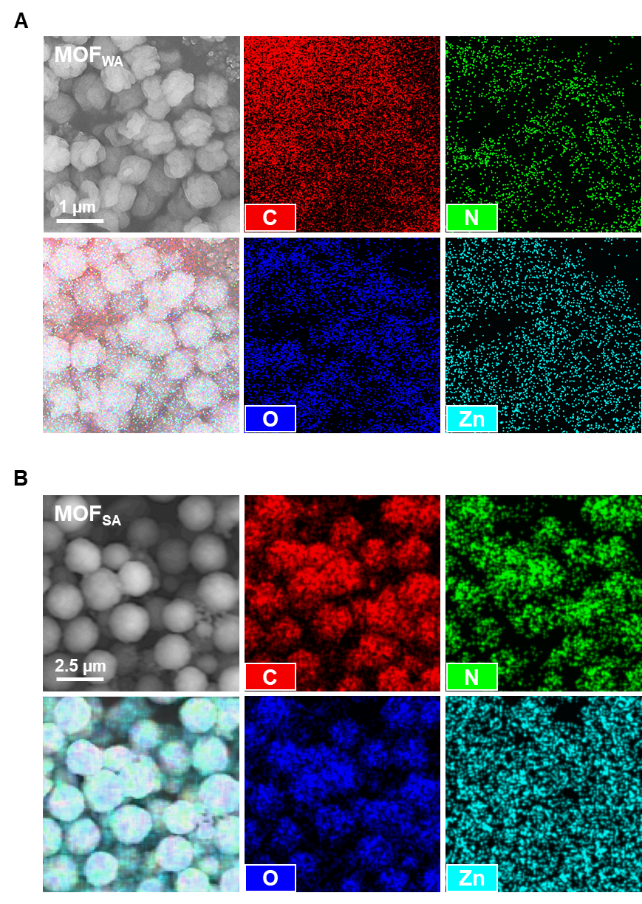


**Figure S1.** EDX mapping (C, N, O, and Zn) of (A) MOF_WA_ and (B) MOF_SA_. MOF_WA_ was synthesized using 12.4 µmol of Zn²⁺ as the raw material, with a molar ratio of Zn²⁺ to imidazole-2-formaldehyde (ICA) of 1:4, and pure water as the solvent. In contrast, MOF_SA_ was generated in the presence of saline.


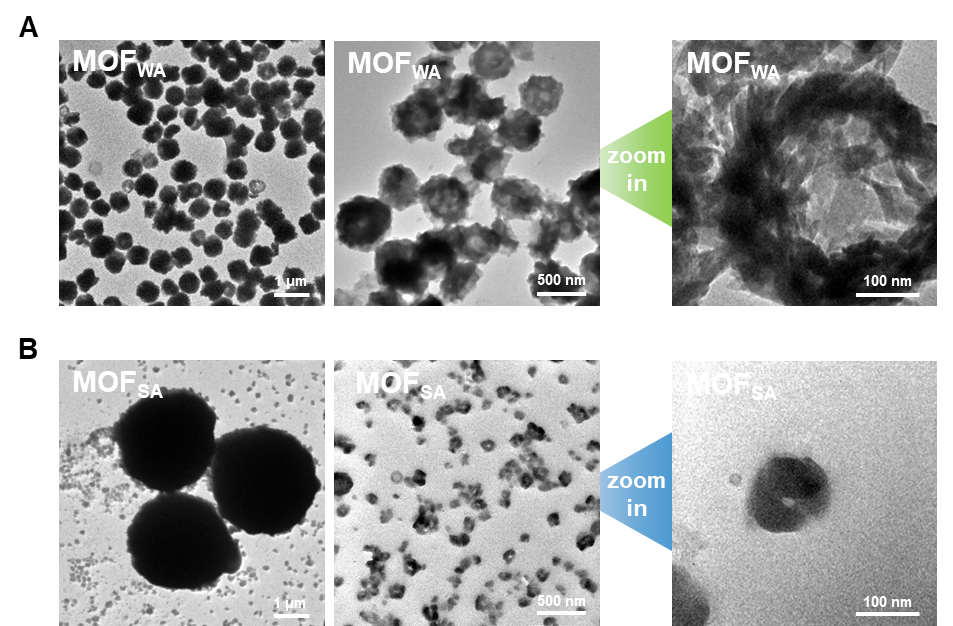


**Figure S2.** TEM images of (A) MOF_WA_ (B) MOF_SA_.


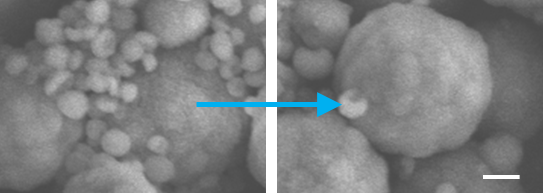


**Figure S3.** SEM images of the MOF_SA_ assembly process. The scale bar is 500 nm.


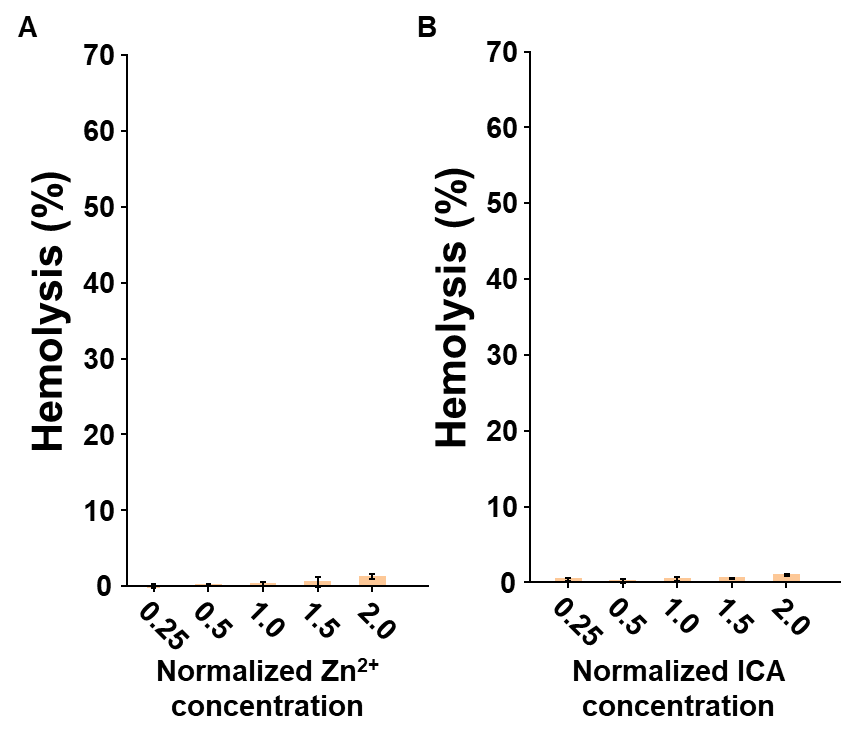


**Figure S4.** Hemolysis assessment of sheep RBCs upon exposure to MOF_SA_ precursors. The concentration in the MOF_SA_ armor synthesis protocol was set at 1.0, corresponding to a Zn^2+^ concentration of 10.55 mM and an ICA concentration of 42.5 mM.


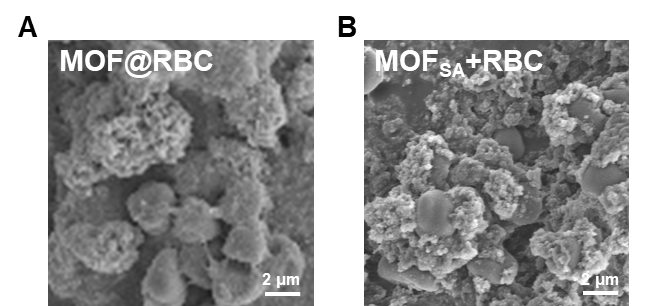


**Figure S5.** SEM images of MOF-armored RBC. (A) MOF@RBC and (B) MOF_SA_+RBC. MOF@RBC was synthesized through an in situ method to load RBCs (5 × 10^8^) into MOF armor. This protective strategy can rapidly create spore-like structures with high efficiency using the MOF platform. MOF_SA_+RBC was prepared using a post-coating method by physically mixing pre-synthesized MOF_SA_ and RBCs.


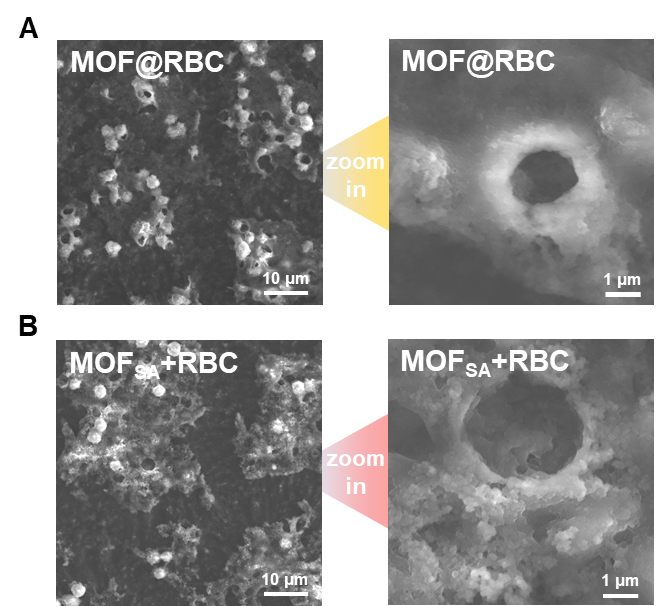


**Figure S6.** SEM images of the MOF shell after washing with pure water to remove loaded RBCs. (A) MOF@RBC and (B) MOF_SA_+RBC.


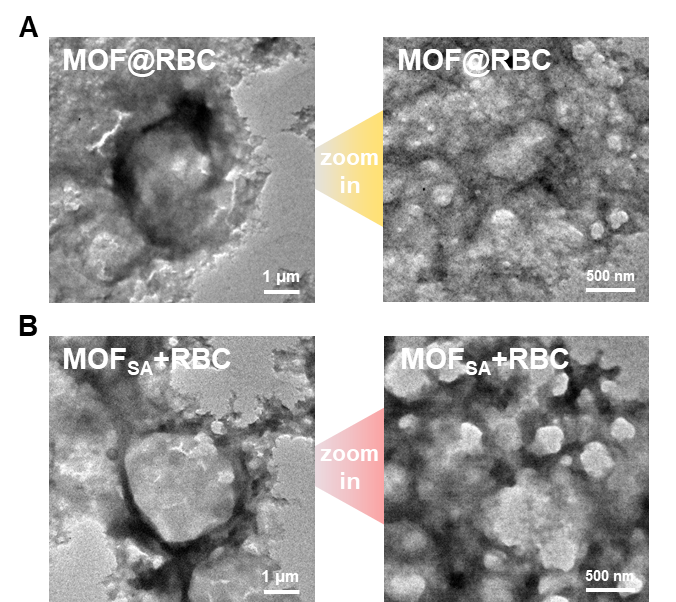


**Figure S7.** TEM images of the MOF shell after washing with pure water to remove loaded RBCs. (A) MOF@RBC and (B) MOF_SA_+RBC.


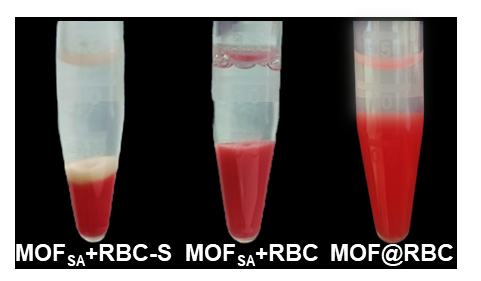


**Figure S8.** Sedimentation of MOF-armored RBC dispersion after resting for 12 h at 4 °C. MOF_SA_+RBC-S was prepared by physically mixing pre-synthesized MOF_SA_ and RBCs for 10 s, distinguishing it from the MOF_SA_+RBC formulation that was prepared for 10 min.


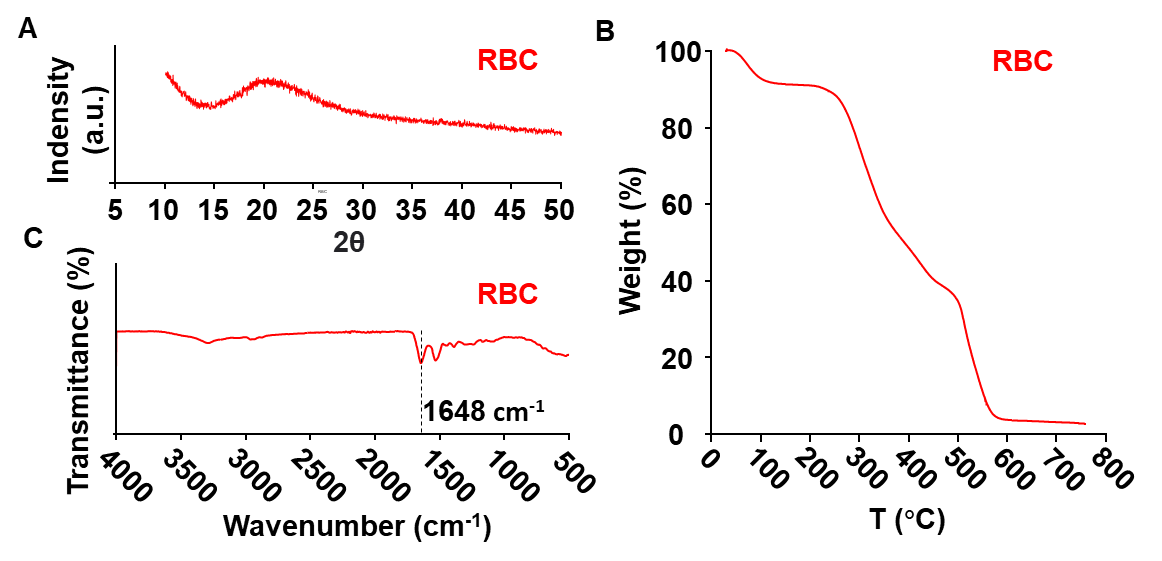


**Figure S9.** Characterization of RBCs. (A) XRD pattern, (B) TGA curve, and (C) FT-IR spectrum.


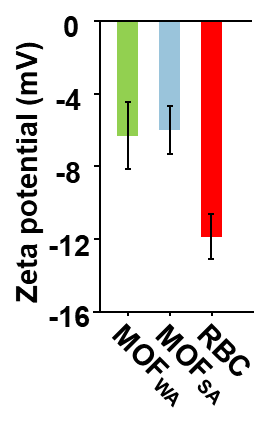


**Figure S10.** Measured zeta potential values for the MOF_WA_, MOF_SA_, and native RBC.


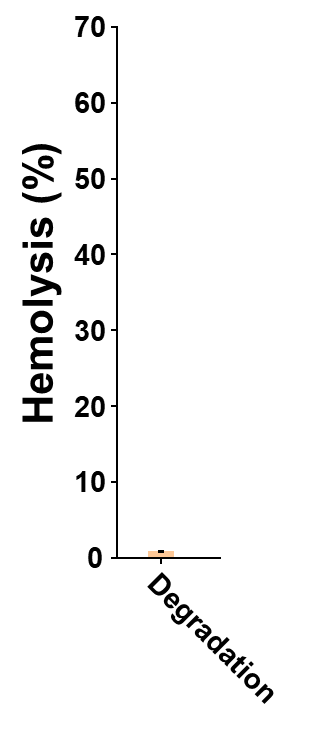


**Figure S11.** Hemolysis assessment of sheep RBCs following a 30-minute exposure to citrate degradation solution.


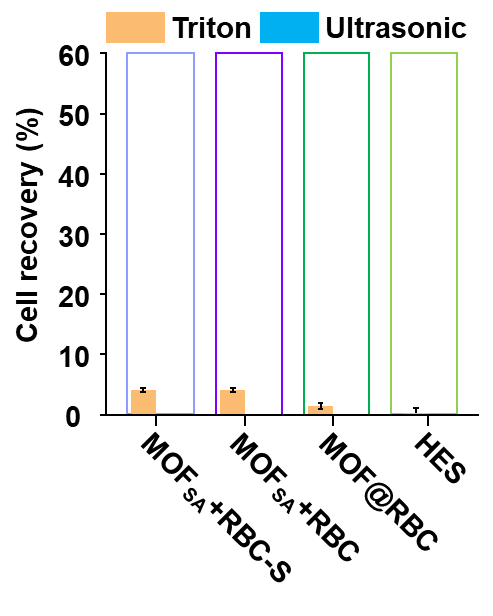


**Figure S12.** Cell recovery rates for the degraded MOF_SA_+RBC-S, MOF_SA_+RBC, and MOF@RBC groups, or HES, following ultrasonic treatment or the addition of Triton-X100 treatment, suggest that the RBCs were completely detached from the MOF shell under this citric acid stepwise degradation protocol.


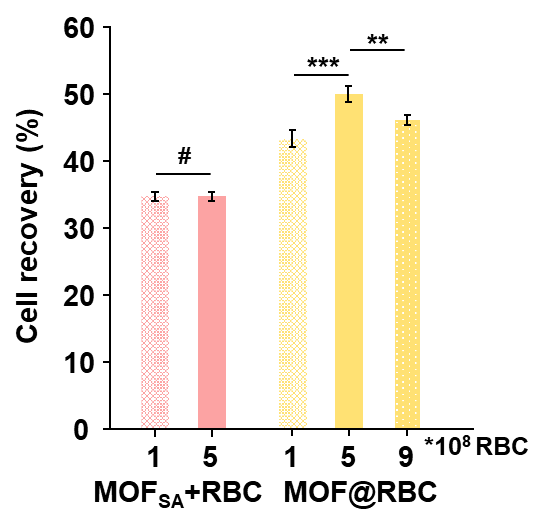


**Figure S13.** Recovery rates of sheep RBCs cryopreserved in MOF structure dispersions under mild thawing conditions at 37 °C. *^*^p* < 0.05; *^**^p* < 0.01; *^#^* indicates no statistically significant difference.


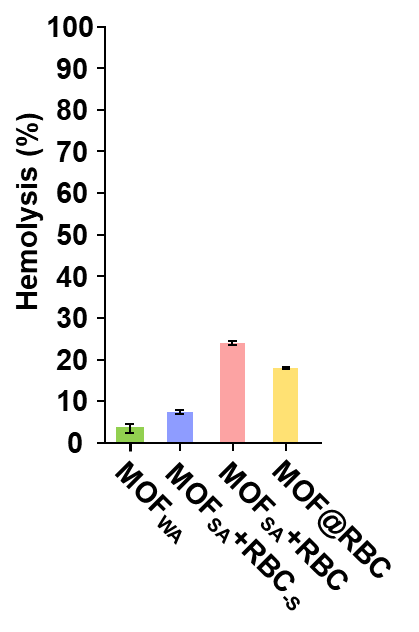


**Figure S14.** Hemolysis rates of sheep RBCs incubated in MOF structures at 4 °C for 24 h, followed by the removal of armor.

**
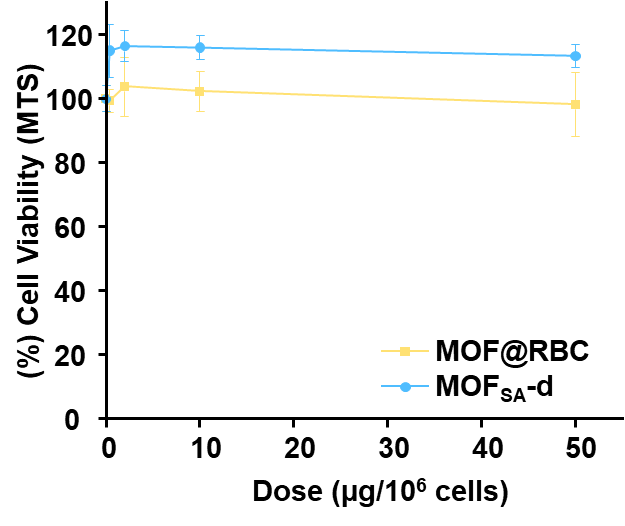
**

**Figure S15.** Cell viability analyses in NIH/3T3. NIH/3T3 were incubated with MOF@RBC (0-50 µg MOF_SA_/10^6^ cells) and the corresponding concentration of MOF_SA_ degradation solution (MOF_SA_-d) for 12 h. Cell viability induced by (A) MOF@RBC and (B) MOF_SA_ degradation solution was determined using an MTS assay (n = 3).


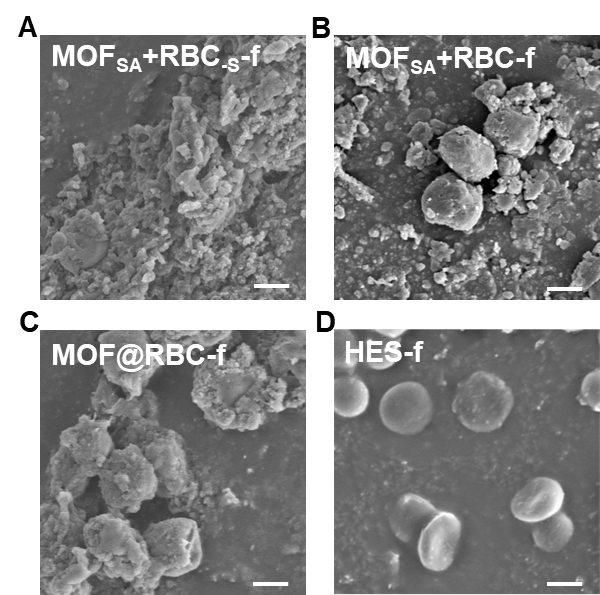


**Figure S16.** SEM images of MOF-armored RBCs (-f indicates that the sample has undergone a treatment of freezing at -80 °C for 24 h and thawing at 37 °C). (A) MOF_SA_+RBC_-S_, (B) MOF_SA_+RBC, (C) MOF@RBC, and (D) HES. The scale bar is 2 µm.


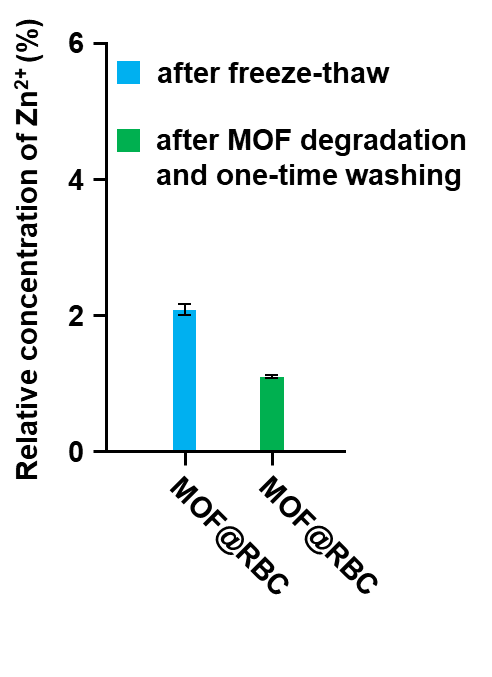


**Figure S17.** Relative concentration of Zn^2+^. The initial Zn^2+^ concentration added under the MOF_SA_ armor synthesis protocol was designated as 100%, corresponding to 10.55 mM.


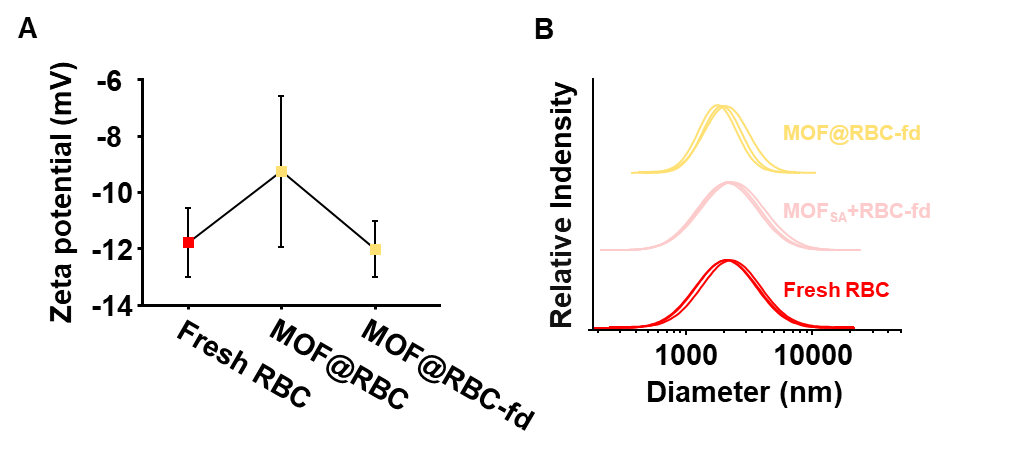


**Figure S18.** (A) The zeta potential and (B) the hydrodynamic sizes of the recovered RBCs in comparison with the fresh RBCs.


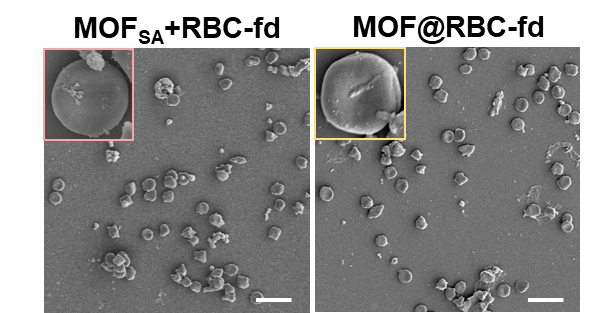


**Figure S19.** SEM images of MOF-armored RBCs (-fd indicates that the sample has undergone freeze-thaw and degradation of MOFs). (A) MOF_SA_+RBC, (B) MOF@RBC. The scale bar is 10 µm.


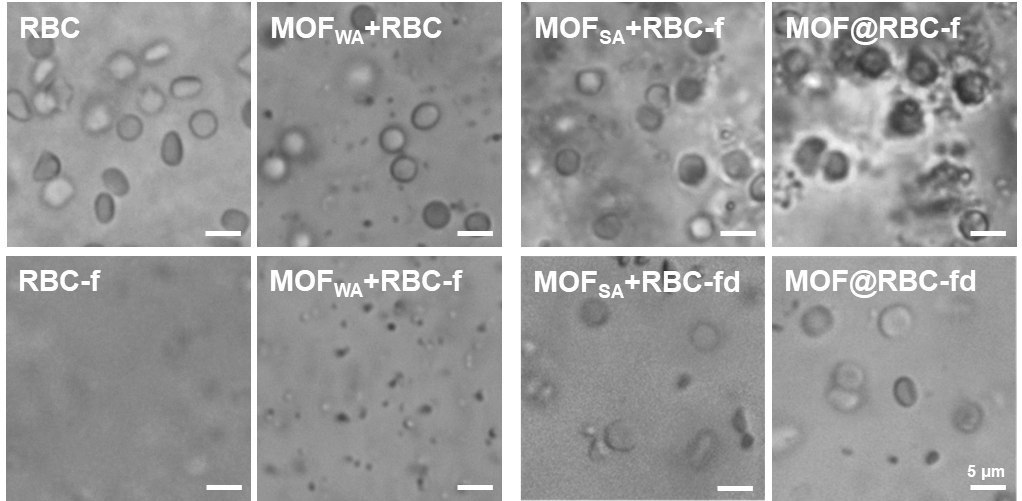


**Figure S20.** Optical microscopy images of MOF-armored RBCs. (-f indicates that the sample has undergone a treatment of freezing at -80 °C for 24 h and thawing at 37 °C; -fd indicates that the sample has undergone freeze-thaw and degradation of MOFs.) The scale bar is 5 µm.


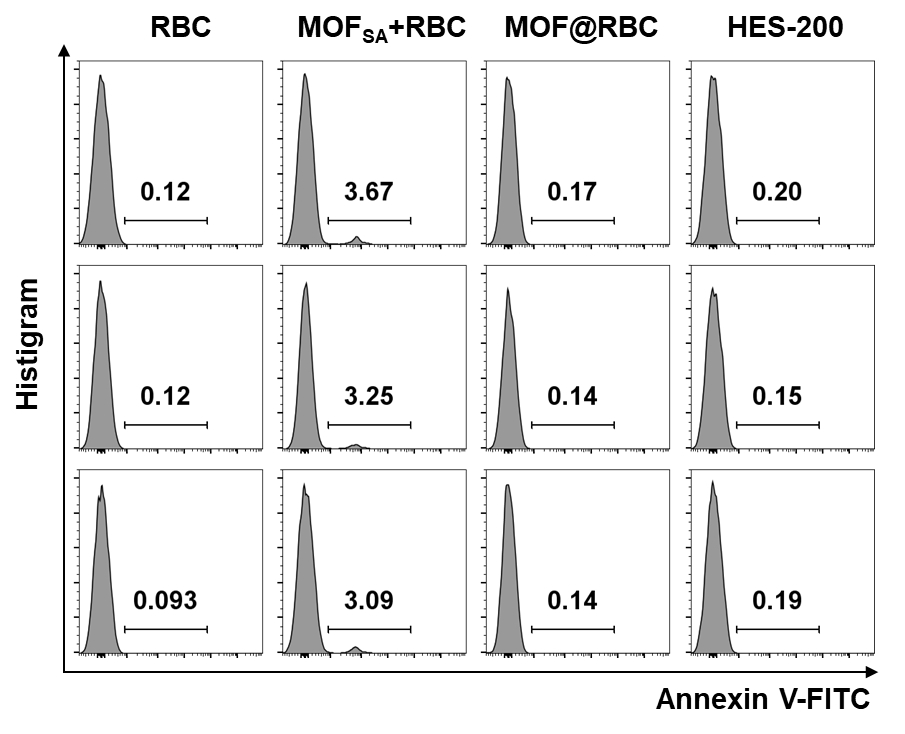


**Figure S21.** The frequency of Annexin V-FITC in native RBCs or RBCs cryopreserved by MOF_SA_+RBC, MOF@RBC, HES.

*
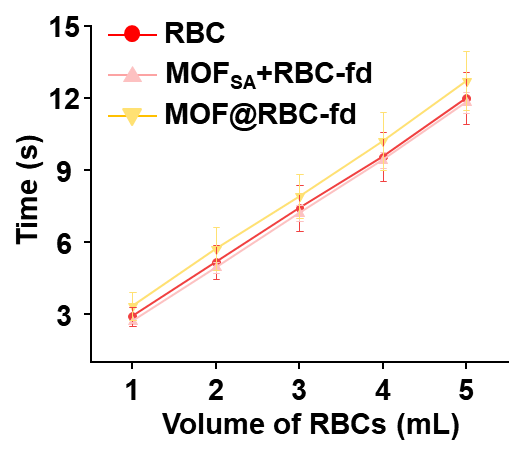
*

**Figure S22.** Assay of RBCs' deformability. The time taken for different volumes of RBC suspension (5×10^7^ RBCs/mL) to pass through a 3 µm filter membrane using the microporous filtration method (n = 3).


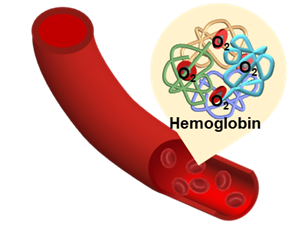


**Figure S23.** Schematic illustration of oxygen binding through hemoglobin in RBCs.


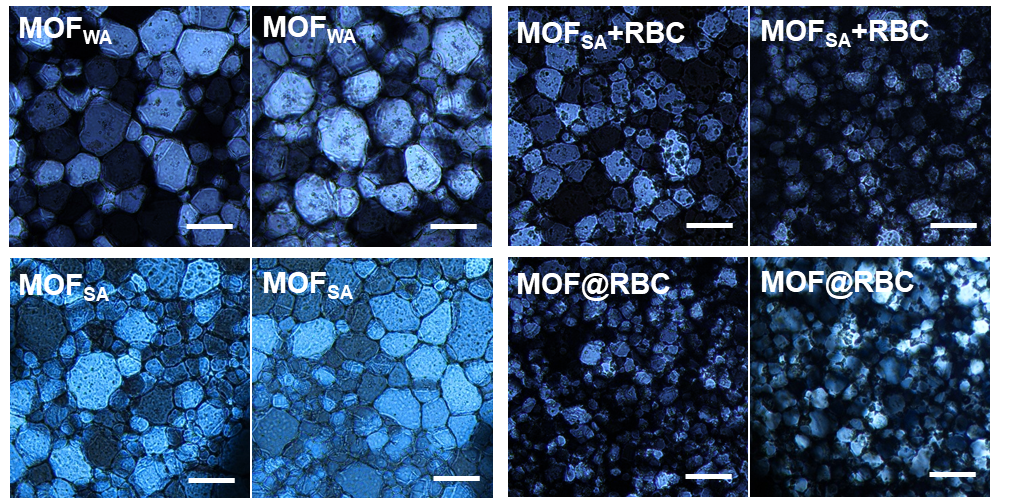
**Figure S24.** Photographs of ice crystals from independent ice wafers, grown in saline dispersions containing various MOF structures, after annealing at -9 °C for 30 min. The scale bar is 100 µm.


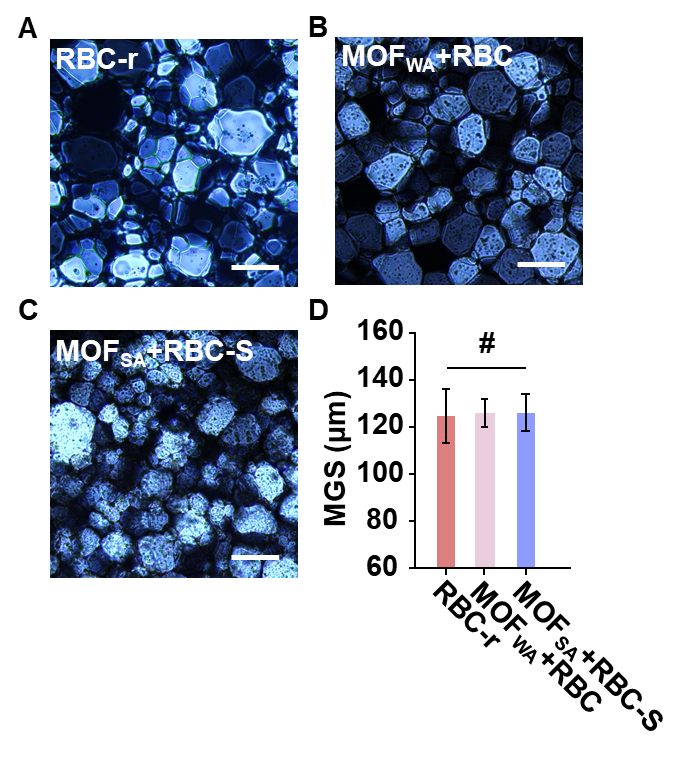


**Figure S25.** Verification of spore-like structures in ice recrystallization inhibition. Polarizing microscope images of ice crystals formed in (A) RBC-r, (B) MOF_WA_+RBC, and (C) MOF_SA_+RBC-S after annealing at −9 °C for 30 min. RBC-r refers to membrane-ruptured RBCs following ultrasonic treatment. MOF_WA_+RBC indicates that the pre-synthesized MOF_WA_ were physically mixed with RBC. (B) Quantitative analysis of the maximum grain size (MGS) of ice crystals. The scale bar is 100 µm. ^#^indicates no statistically significant difference.

**
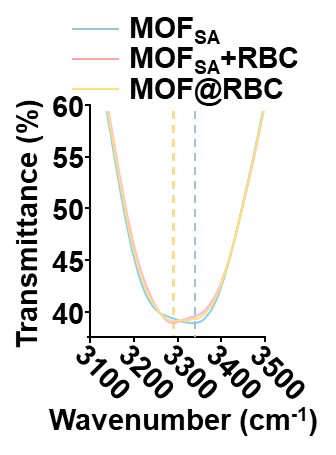
**

**Figure S26.** The attenuated total reflection Fourier-transform infrared (ATR-FTIR) spectrum of MOF_SA_, MOF@RBC, and MOF_SA_+RBC was analyzed in the liquid phase. The material's volume was concentrated 33 times following the synthesis protocol using 12.4 µmol of Zn²⁺ as the raw material, with a molar ratio of Zn²⁺ to imidazole-2-formaldehyde (ICA) of 1:4, and saline as the solvent.

**
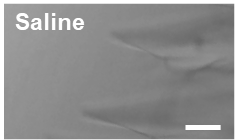
**

**Figure S27.** The directional ice growth microscopy (DIGM) image of saline reveals a sawtooth-like morphology at the ice-water interface, resulting from rapid growth occurring at a rate of 15 μm/s. This value is derived from the average measurement over a 150 μm distance. The scale bar is 50 µm.

**
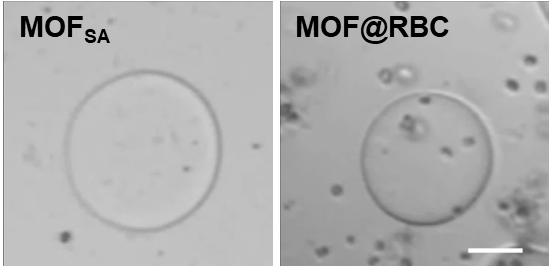
**

**Figure S28.** Shape of single ice crystals grown in MOF_SA_ and MOF@RBC dispersions at -0.2 °C. The scale bar is 20 µm.

**Table S1.** Elemental analysis results from the elemental analyzer for MOF_WA_ and MOF_SA_

| **Element** | **Atom_MOFWA_ (%)** | **Atom_MOFSA_ (%)** |
| --- | --- | --- |
| C | 69.2 | 47.6 |
| N | 11.8 | 23.2 |
| O | 16.0 | 23.9 |
| Zn | 3.0 | 5.3 |

| **Element ratio** | **MOF_WA_** | **MOF_SA_** |
| --- | --- | --- |
| **Zn: N** | **4: 1** | **4: 1** |

**Table S2.** Osmotic pressure of saline and citric acid phosphate buffer (pH 6.25).

| **Sample ID** | **Osmotic pressure (mmol/kg)** |
| --- | --- |
| **Saline** | **279 ± 4** |
| **Citric acid phosphate buffer (pH 6.25)** | **287 ± 3** |

**Table S3.** Degradation rates of MOF_WA_, MOF_SA_, and MOF@RBC under a stepwise addition protocol with a citric acid phosphate buffer at pH 6.25.

| **Sample ID** | **Degradation rate (%)** |
| --- | --- |
| **MOF_WA_** | **91 ± 2** |
| **MOF_SA_** | **91 ± 4** |
| **MOF@RBC** | **86 ± 3** |
